# Transforming Spinal Muscular Atrophy: From Pivotal Trials to Real-World Evidence and Future Therapeutic Frontiers in Types 1 and 2

**DOI:** 10.3390/biomedicines13081939

**Published:** 2025-08-08

**Authors:** Andrej Belančić, Patrick Castillo Eustaquio, Elvira Meni Maria Gkrinia, Valentino Rački, Kristina Pilipović, Dinko Vitezić

**Affiliations:** 1 Department of Basic and Clinical Pharmacology and Toxicology, Faculty of Medicine, University of Rijeka, 51000 Rijeka, Croatia; kristina.pilipovic@uniri.hr (K.P.); dinko.vitezic@uniri.hr (D.V.); 2 Independent Researcher, Manila 1000, Philippines; eustaquiopatrickmd@gmail.com; 3 Independent Researcher, 11527 Athens, Greece; 4 Department of Neurology, Clinical Hospital Centre Rijeka, 51000 Rijeka, Croatia; valentino.racki@uniri.hr

**Keywords:** spinal muscular atrophy, SMA, disease-modifying drugs, nusinersen, risdiplam, onasemnogene abeparvovec, real-world evidence

## Abstract

Spinal muscular atrophy (SMA) is a rare, autosomal recessive neuromuscular disorder and a leading genetic cause of infant mortality. The past decade has witnessed a paradigm shift in SMA management with the advent of disease-modifying drugs (DMDs). This narrative review aims to (i) summarize pivotal randomized controlled trials (RCTs) that led to the approval of DMDs for SMA Types 1 and 2; (ii) synthesize real-world evidence on their safety and effectiveness; and (iii) explore emerging therapeutic frontiers, including gene modifiers, predictive biomarkers, prenatal interventions, and combination strategies. Pivotal RCTs and real-world studies demonstrate that onasemnogene abeparvovec (a single-dose gene therapy), nusinersen (an intrathecal antisense oligonucleotide), and risdiplam (an oral SMN2 splicing modifier) each significantly improve survival and motor function milestones compared to natural history in Type 1 and Type 2 SMA, with the majority of treated patients achieving independent sitting and prolonged ventilator-free survival, while safety profiles are generally manageable and distinct for each therapy. Similar outcomes have been demonstrated for presymptomatic patients with SMA. The introduction of DMDs has transformed the prognosis of SMA, particularly for early-onset forms, with robust evidence supporting their efficacy and safety. Continued real-world monitoring and exploration of adjunctive therapies are essential to optimize outcomes across the SMA setting and address unmet needs in non-responders and older patients.

## 1. Introduction

Spinal muscular atrophy (SMA) is a rare autosomal recessive neuromuscular disorder characterized by progressive degeneration of alpha motor neurons in the anterior horn of the spinal cord, resulting in symmetric muscle weakness and atrophy. SMA has an estimated prevalence of 1 to 2 per 100,000 individuals and an incidence of approximately 1 in 10,000 live births; it represents one of the most common genetic causes of infant mortality. The underlying genetic defect is typically a homozygous deletion or mutation in the SMN1 gene, leading to insufficient levels of survival motor neuron (SMN) protein, a critical factor for motor neuron maintenance and function [1,2].

Disease severity is partially modulated by the copy number of the SMN2 gene, a paralog that undergoes alternative splicing and produces only a fraction of functional SMN protein. The number of SMN2 exon 7 copies has emerged as a key prognostic biomarker and is increasingly used to predict therapeutic response to disease-modifying drugs (DMDs) [3,4]. Based on age at symptom onset, maximum motor milestone achieved, and overall disease severity, SMA is classified into five clinical types, which are further divided into subtypes, with Type 1 being the most common. Thus, the phenotype of SMA may range from severe neonatal presentations with early mortality (Type 0) to adult-onset forms characterized by mild proximal muscle weakness (Type 4) [5,6].

**Figure 1:**
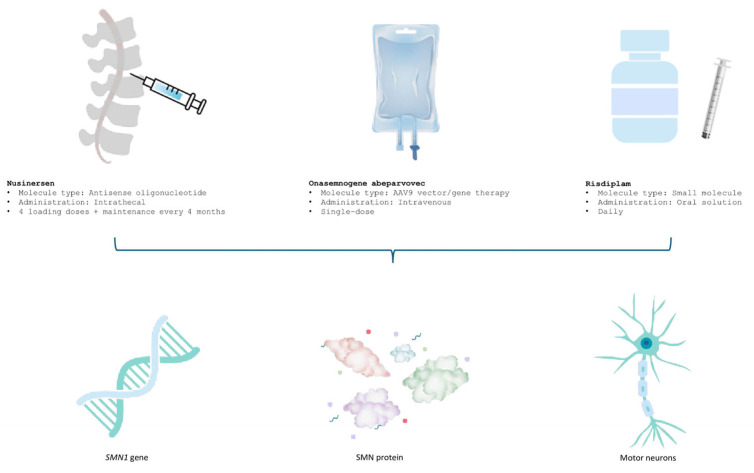
Mechanisms of action of approved SMA treatments.

Given the multisystemic burden of the disease, optimal care necessitates a coordinated multidisciplinary approach encompassing neurology, respiratory medicine, orthopedics, rehabilitation, and nutritional support [7,8]. Over the past decade, the therapeutic landscape of SMA has undergone a paradigm shift with the emergence of DMDs, which target the underlying genetic defect and have shown transformative benefits in clinical trials. These therapies have redefined prognosis and functional outcomes, particularly in early-onset forms [9,10]. Figure 1 illustrates the distinct mechanisms of action of the three approved SMA treatments, highlighting their targets and therapeutic approaches for improved clinical understanding. The long-term real-world effectiveness, safety, and differential impact across SMA phenotypes remain areas of active investigation.

### Search Strategy

This narrative review synthesized evidence on the clinical efficacy, effectiveness, and safety of treatments for SMA. The search was performed using the following search strings: ((“genetic based therapy”) OR (“splicing modifi*”) OR (“gene replacement”) OR (“gene therapy”) OR (“SMN 2”) OR (nusinersen) OR (spinraza) OR (“onasemnogene abeparvovec”) OR (zolgensma) OR (risdiplam) OR (evrysdi)) AND ((spinal muscular atrophy OR SMA)). Search limits in PubMed included filtering for “clinical study”, “clinical trial”, “multicenter study”, and “observational study”. Eligible studies were selected based on the criteria included in Table 1. Studies were excluded if they focused solely on preclinical data, lacked efficacy/effectiveness outcomes, or were not published in English.

The review employed a narrative synthesis methodology to integrate findings from diverse study designs. This approach allowed for a comprehensive interpretation of the evidence while highlighting variations in study contexts and methodologies. Insights were framed within the broader context of evolving SMA treatment practices. By combining systematic rigor with narrative flexibility, this review aims to provide a state-of-the-art summary of SMA treatment efficacy and effectiveness in clinical practice settings [11].

This narrative review aims to (i) summarize the pivotal randomized controlled trials that led to the approval of all disease-modifying therapies for SMA Types 1 and 2; (ii) synthesize real-world evidence on their safety and effectiveness; and (iii) explore future therapeutic directions, including gene modifiers, predictive biomarkers, prenatal interventions, combination strategies, and glial modulation as potential adjunctive approaches.

## 2. Results

### 2.1. RCT Data

#### 2.1.1. Nusinersen: Pivotal RCT Evidence

Nusinersen, an intrathecally administered antisense oligonucleotide (ASO), was the first DMD approved for the treatment of SMA, receiving European Medicines Agency (EMA) approval in May 2017. The compound exerts its therapeutic effect by binding to the intronic splice silencing site (ISS-N1) in intron 7 of the *SMN2* pre-mRNA. This interaction displaces repressive splicing factors, promoting exon 7 inclusion in *SMN2* transcripts and restoring production of full-length SMN protein [12].

The phase 3 ENDEAR trial evaluated the clinical efficacy of nusinersen in 121 infants with symptomatic SMA (symptom onset before 6 months of age), all ≤7 months at screening. Participants were randomized 2:1 to receive nusinersen or a sham procedure, with treatment durations ranging from 6 to 442 days. The cohort was genetically homogeneous, with 99% of participants carrying two *SMN2* copies, consistent with Type 1 SMA. The median age of symptom onset was 6.5 weeks in the nusinersen group and 8 weeks in controls; median age at first dose was 164.5 days vs. 205 days, respectively. Baseline characteristics were broadly similar, though the nusinersen group showed higher rates of paradoxical breathing (89% vs. 66%), pneumonia or respiratory symptoms (35% vs. 22%), feeding difficulties (51% vs. 29%), and respiratory support (26% vs. 15%). In the final analysis, participants treated with nusinersen showed statistically and clinically significant improvements. A motor milestone response was achieved in 51% of nusinersen-treated infants, compared to 0% in the sham group (*p* < 0.0001). The primary endpoint—time to death or permanent ventilation (≥16 h/day for >21 consecutive days or tracheostomy, excluding acute reversible causes)—also favored nusinersen. Improvement in Children’s Hospital of Philadelphia Infant Test of Neuromuscular Disorders (CHOP-INTEND) scores (≥4-point gain from baseline) was significantly more common in the treatment group. Among patients requiring permanent ventilation, 25% (18/72) in the nusinersen group and 32% (12/37) in the sham group met the CHOP-INTEND criteria. Of those, six patients (33%) treated with nusinersen achieved a motor milestone response, while none in the sham group did.

Long-term efficacy outcomes were assessed in the SHINE extension study (CS11), which enrolled 89 participants from ENDEAR (nusinersen: n = 65; sham: n = 24). Patients who received nusinersen continuously were treated for 6 to 3043 days (median 2443), while those initiating treatment after the sham period received it for 65 to 2520 days (median 2090). Motor function improvements persisted in both cohorts, with the greatest benefit among those treated earlier. The majority of patients were alive at their last visit following nusinersen initiation [12,13].

The CHERISH trial (Study CS4) was a phase 3, randomized, double-blind, sham-controlled trial that enrolled 126 symptomatic patients with later-onset SMA (symptom onset after 6 months of age), reflecting a population likely to develop Type 2 or 3 SMA. Participants were randomized 2:1 to receive nusinersen (3 loading doses followed by a maintenance dose every 6 months) or a sham procedure, with treatment durations ranging from 324 to 482 days. The median age at screening was 3 years, and the median age of SMA symptom onset was 11 months. The majority (88%) had three *SMN2* copies; 8% had two, 2% had four, and 2% had unknown copy numbers. All patients had achieved independent sitting, but none had achieved independent walking. Baseline motor scores were consistent: mean Hammersmith Functional Motor Scale—Expanded (HFMSE) was 21.6, and Revised Upper Limb Module (RULM) was 19.1. An imbalance was observed in two motor milestones: standing without support (13% in the nusinersen group vs. 29% in sham) and walking with support (24% vs. 33%). The intention-to-treat (ITT) analysis (nusinersen: n = 84; sham: n = 42) showed a statistically significant improvement in HFMSE score from baseline to Month 15 among those treated with nusinersen. Among patients with observed data at Month 15, 73% of those treated with nusinersen demonstrated improvement in HFMSE vs. 41% in the sham group. Conversely, worsening of motor function was more common in the sham group (44%) than in those receiving nusinersen (23%). Results remained consistent in multiple imputation sensitivity analyses. Secondary endpoints, including additional motor function metrics and World Health Organization (WHO) motor milestone achievement, also favored nusinersen. Early treatment initiation correlated with both earlier and greater motor gains, although patients treated later still benefited over controls.

Following the completion of the CHERISH trial, 125 participants (nusinersen: n = 83; sham: n = 42) entered the SHINE extension study, which is an open-label trial that evaluated the long-term clinical efficacy and safety of nusinersen. In both groups, stabilization or further improvement in motor function was observed, with the greatest benefit again seen in those who began nusinersen earlier [12,14].

The most common adverse events (AEs) associated with the administration of nusinersen by lumbar puncture were headache, vomiting, and back pain [12]. There were no safety signals or serious AEs reported.

#### 2.1.2. Risdiplam: Pivotal RCT Evidence

Risdiplam, a once-daily oral small molecule, is an SMN2 pre-mRNA splicing modifier developed to treat 5q-associated SMA caused by mutations in the SMN1 gene. By promoting the inclusion of exon 7 in SMN2 transcripts, treatment with risdiplam increases production of full-length, functional SMN protein, thereby addressing the underlying cause of SMA pathophysiology-progressive motor neuron degeneration due to SMN protein deficiency. Risdiplam received EMA approval in March 2021 based on the pivotal FIREFISH and SUNFISH clinical trials, which established its efficacy and safety in both infantile-onset (Type 1) and later-onset (Types 2 and 3) SMA across a broad pediatric and young adult population [15].

The FIREFISH study (BP39056) was a two-part, open-label trial evaluating the efficacy, safety, pharmacokinetics (PK), and pharmacodynamics (PD) of risdiplam in symptomatic infants with genetically confirmed Type 1 SMA and two copies of the SMN2 gene. Part 1 was a dose-finding phase, while Part 2 assessed efficacy at the therapeutic dose. Patients from Part 1 were not enrolled in Part 2. In FIREFISH Part 2, 41 infants were enrolled with a median age at symptom onset of 1.5 months (range 1.0–3.0) and a median age at enrolment of 5.3 months (range 2.2–6.9). The cohort was 54% female, 54% Caucasian, and 34% Asian. At baseline, the median CHOP-INTEND score was 22.0 (range 8.0–37.0), and the median Hammersmith Infant Neurological Examination—Section 2 (HINE-2) score was 1.0 (range 0.0–5.0). After 12 months of treatment, 29% of patients achieved the primary endpoint of sitting without support for at least 5 s, measured by Item 22 of the Bayley Scales of Infant and Toddler Development, Third Edition (BSID-III). By Month 24, 44% of infants could sit unsupported for 30 s (BSID-III Item 26), 80.5% were able to roll, and 27% achieved a standing response, with 12% supporting weight and 15% standing with support. In contrast, untreated infants with Type 1 SMA would be expected neither to sit independently nor survive beyond 14 months of age without permanent ventilation, with survival rates estimated at only 25% [15,16].

Additional evidence was provided by FIREFISH Part 1, which enrolled 21 infants with a median enrolment age of 6.7 months (range 3.3–6.9) and a median time from symptom onset to treatment initiation of 4.0 months (range 2.0–5.8). Seventeen infants received the therapeutic dose selected for Part 2. At 12 months, 41% (7/17) of these patients could sit independently for at least 5 s; this proportion rose to 59% (10/17) by 24 months. Importantly, 90% (19/21) of all patients in Part 1 were alive and free of permanent ventilation at 12 months and had reached 15 months of age or older. After a minimum of 33 months of treatment, 81% (17/21) remained alive and event-free, reaching a median age of 41 months (range 37–53). Three patients died during treatment, and one additional patient died 3.5 months after treatment discontinuation [15,16].

Further evidence on FIREFISH Part 1 was published by Baranello et al., where they evaluated the safety, PK, PD, and dose selection for risdiplam among infants aged 1–7 months with genetically confirmed Type 1 SMA, two SMN2 copies, and who had not previously received other SMN-targeted or any other gene therapy. Among the 21 infants who were enrolled, 4 were assigned to a low-dose cohort (0.08 mg/kg/day) and 17 were assigned to a high-dose cohort (0.2 mg/kg/day). At baseline, all patients were non-sitters, with a median CHOP-INTEND score of 24, and almost a quarter required respiratory support. After 12 months, the treatment resulted in dose-dependent increases in blood SMN protein levels and functional improvements. Particularly, 7 infants in the high-dose group were able to sit independently, 11 achieved CHOP-INTEND scores of at least 30, and 86% could feed orally. However, serious adverse events (SAEs) occurred in almost half of the patients, the most common of which were respiratory infections, while there were four deaths due to complications. Nonetheless, a favorable benefit–risk ratio was demonstrated for treatment with risdiplam. The 0.2 mg/kg/day dose was selected for efficacy evaluation for FIREFISH Part 2 [17].

SUNFISH (BP39055) was a multicentre, two-part study evaluating risdiplam in patients aged 2 to 25 years with non-ambulant Type 2 or 3 SMA. Part 1 was an exploratory dose-finding study, while Part 2 was a randomized, double-blind, placebo-controlled phase that served as the confirmatory efficacy trial. In SUNFISH Part 2, 180 patients were randomized in a 2:1 ratio to receive risdiplam or placebo, stratified by age groups (2–5, 6–11, 12–17, and 18–25 years). The median age at enrolment was 9.0 years (range 2–25), with a median interval from symptom onset to treatment initiation of 102.6 months (range 1–275). The cohort included 51% females, 67% Caucasians, and 19% Asians. At baseline, scoliosis was present in 67% of patients (73% in the placebo group vs. 63% in the risdiplam group), including 32% with severe scoliosis. Mean baseline scores were 46.1 for the Motor Function Measure-32 (MFM32) and 20.1 for RULM. The primary endpoint-change in MFM32 scores from baseline to Month 12 demonstrated a clinically meaningful and statistically significant benefit in the risdiplam group compared to placebo. Among the 117 patients who continued risdiplam beyond 12 months, motor function improvement was maintained through Month 24, with a mean change in MFM32 of 1.83 (95% confidence interval [CI]: 0.74–2.92) and a mean RULM change of 2.79 (95% CI: 1.94–3.64) [15,18].

Findings from SUNFISH Part 1 supported these outcomes. A total of 51 patients with SMA Types 2 and 3—including seven ambulant individuals—were treated over one year. A clinically meaningful motor function improvement was observed with a mean MFM32 score increase of 2.7 points (95% CI: 1.5–3.8) at 12 months. This improvement was sustained at two years (mean change 2.7; 95% CI: 1.2–4.2) [15,18].

In clinical trials of risdiplam, the most frequently reported AEs among patients with infantile-onset SMA were pyrexia (54.8%), rash (29.0%), and diarrhea (19.4%). In patients with later-onset SMA, the most common AEs were pyrexia (21.7%), headache (20.0%), diarrhea (16.7%), and rash (16.7%). These AEs exhibited no consistent temporal or clinical pattern and typically resolved without necessitating treatment discontinuation in both infantile-onset and later-onset cohorts [15].

#### 2.1.3. Onesamnogene Abeparvovec: Pivotal RCT Evidence

Onasemnogene abeparvovec, an intravenously administered gene replacement therapy, was conditionally approved by the EMA in May 2020, with full marketing authorization granted in May 2022. This approval was supported by pivotal efficacy and safety data from the START and STR1VE clinical trials. The therapy is specifically designed to address the monogenic cause of SMA by delivering a functional copy of the *SMN1* gene. Onasemnogene abeparvovec consists of a non-replicating recombinant adeno-associated virus serotype 9 (AAV9) vector containing a self-complementary, double-stranded cDNA sequence encoding the human SMN protein, driven by a cytomegalovirus-enhanced chicken-β-actin hybrid promoter. Following intravenous administration, the AAV9 capsid efficiently crosses the blood–brain barrier and transduces motor neurons, enabling sustained SMN protein production. The introduced *SMN1* gene remains episomal within the nuclei of post-mitotic target cells, supporting durable expression without integrating into the host genome. The AAV9 vector used is non-pathogenic in humans, and its biodistribution and cellular tropism have been well characterized in both non-clinical and human studies. This continuous and robust SMN expression in transduced cells forms the therapeutic basis for improving motor neuron survival and function in SMA patients [19].

The efficacy and safety of onasemnogene abeparvovec administered as a single intravenous infusion at a therapeutic dose of 1.1 × 10^14^ vector genomes per kilogram (vg/kg) have been evaluated in several pivotal clinical trials involving infants with Type 1 SMA carrying two copies of *SMN2* [20,21,22].

The Phase 3 AVXS-101-CL-303 study was an open-label, single-arm trial enrolling 22 infants with Type 1 SMA, none of whom required non-invasive ventilatory support or enteral feeding at baseline. Participants were younger compared to other cohorts, with a mean age of 3.7 months (range 0.5–5.9), and demonstrated a mean baseline CHOP-INTEND motor function score of 32.0 (range 18.0–52.0). Following treatment, 21 of 22 participants survived without permanent ventilation to at least 10.5 months of age. At 14 months, 90.9% (20/22; 95% CI: 79.7–100.0) remained event-free (alive and free of permanent ventilation), thus meeting one of the study’s co-primary efficacy endpoints, which persisted unchanged at 18 months. The second co-primary endpoint, independent sitting for at least 30 s at 18 months, was achieved by 59.1% (13/22, *p* < 0.0001). Among the 14 participants who attained this milestone at any visit, the median age was 12.6 months (range 9.2–18.6). Although three participants (13.6%) failed to reach any motor milestones, and another three (13.6%) achieved only head control by 18 months, one participant (4.5%) attained walking with assistance by 12.9 months. At 18 months, 18 participants remained completely free of ventilatory support. Motor function improvements were significant: 95.5% achieved CHOP-INTEND scores ≥ 40, 63.6% reached ≥50, and 40.9% attained ≥58—levels rarely observed in untreated infants with Type 1 SMA. Interestingly, motor milestone acquisition did not always correlate directly with CHOP-INTEND scores, as some milestones were achieved even after score plateaus [21,22].

Supporting these results, the phase 1 AVXS-101-CL-101 study treated 12 infants aged 0.9 to 7.9 months (weight range 3.6–8.4 kg) with a single intravenous infusion of onasemnogene abeparvovec. All participants survived without the need for permanent ventilatory support (event-free) at 14 months, compared to only 25% in an untreated natural history cohort. At 24 months post-dose, all 12 remained event-free, contrasting starkly with the less than 8% event-free survival reported historically in untreated infants. Regarding motor milestones, 10 of 12 participants could sit independently for at least 10 s, 9 sustained independent sitting for 30 s or more, and 2 attained standing and walking without assistance by 24 months. One participant did not achieve head control, the highest milestone before age two. Ten participants entered a long-term follow-up study extending to 6.6 years post-treatment, with all of the patients being alive and free of permanent ventilation as of 23 May 2021. During follow-up visits, participants éther maintained previous milestones or attained new ones such as supported sitting, standing with assistance, and independent walking. Five participants received concomitant nusinersen or risdiplam at some point, complicating attribution of all gains to gene therapy alone. Nonetheless, two participants who were not treated with an additional DMD still acquired new milestones, supporting the sustained therapeutic impact of onasemnogene abeparvovec [19,21].

The Phase 3 AVXS-101-CL-302 enrolled 33 infants with Type 1 SMA, including a more clinically heterogeneous population, with 27.3% requiring ventilatory support and 27.3% needing feeding support at baseline. The mean age at dosing was 4.1 months (range 1.8–6.0), with a mean baseline CHOP-INTEND score of 27.9 (range 14–55). One participant was dosed outside the protocol-defined age range and excluded from the ITT analysis, resulting in 32 evaluable participants for efficacy outcomes. Among these, 31 (96.9%) survived event-free for at least 14 months, meeting the key secondary endpoint. One participant (3%) died due to disease progression. The primary endpoint of independent sitting for at least 10 s by 18 months was achieved by 14 participants (43.8%) in the ITT sample, with a median milestone acquisition age of 15.9 months (range 7.7–18.6). Notably, one participant attained multiple advanced motor milestones—including crawling, standing with assistance, standing independently, walking with assistance, and walking independently—by 18 months. CHOP-INTEND scores improved substantially: 72.7% achieved scores ≥ 40, 42.4% ≥ 50, and 9.1% ≥ 58. These outcomes markedly surpass the natural history of untreated Type 1 SMA, where CHOP-INTEND scores ≥ 40 are exceedingly rare, underscoring the robust efficacy of onasemnogene abeparvovec [19,22].

The safety profile of onasemnogene abeparvovec was established through data collected from 99 participants treated at the recommended dose of 1.1 × 10^14^ vg/kg across five open-label clinical studies. The most commonly reported AEs included elevated hepatic enzymes (24.2%), hepatotoxicity (9.1%), vomiting (8.1%), thrombocytopenia (6.1%), increased troponin levels (5.1%), and pyrexia (5.1%) [19].

A single-center, open-label phase 1/2 trial by Mendell et al. examined the safety and efficacy of single-dose intravenous onasemnogene abeparvovec in infants with Type 1 SMA. Genetically confirmed patients with homozygous *SMN1* deletions and two copies of *SMN2* were enrolled. Between May 2014 and December 2015, 15 participants were enrolled at the Nationwide Children’s Hospital in Ohio, with a median age at treatment of 3.4 months (range 0.9–7.9 months); 53% were female. All participants showed early symptom onset and profound motor weakness at baseline. Three participants received a low-dose (6.7 × 10^13^ vg/kg) and 12 received a high-dose (2.0 × 10^14^ vg/kg) regimen. Data on AEs were collected using Common Terminology Criteria for Adverse Events (CTCAE), time to death or permanent ventilation, and assessment of motor function, particularly milestones using WHO criteria and Bayley scales and function scores using CHOP-INTEND. At median follow-up of 25.7–30.8 months, all 15 patients were alive and event-free, compared to 8% in the control group. Among the high-dose group, the mean CHOP-INTEND scores increased by 9.8 points at 1 month (*p* < 0.001) and 15.4 points at 3 months (*p* < 0.001), with continuous improvement over time. Almost all participants (11/12) achieved unassisted sitting, and some attained rolling, standing, and walking independently. More importantly, these milestones were not observed in the control group. Two participants experienced transient grade 4 elevations in liver transaminases, which were managed with prednisolone. Nonetheless, there were no treatment discontinuations due to AEs [21].

The SPR1NT trial by Strauss et al. (2022) was a multi-center, phase 3, single-arm clinical trial conducted to evaluate the efficacy and safety of onasemnogene abeparvovec in presymptomatic infants. Between April 2017 and December 2020, infants with genetically confirmed *SMN1* deletions and two *SMN2* copies, with no signs and symptoms of neuromuscular disease, were enrolled and received treatment by six weeks of age. Motor milestones were assessed using the BSID and WHO-MGRS criteria, and motor function was assessed using CHOP-INTEND scores. A total of 14 participants were studied, all with Type 1 SMA, 57% male, with a mean age of 20.6 days at dosing. Independent sitting for ≥30 s by 18 months was achieved by all 14 participants, compared to the Pediatric Neuromuscular Clinical Research (PNCR) cohort (*p* < 0.001). All participants were alive and free from a ventilator at 14 months, compared to 26% in the PNCR cohort (*p* < 0.001). Further, 93% maintained ≥3rd WHO percentile through 18 months without feeding support. Furthermore, 64% were able to walk independently, and 79% were able to stand alone by 18 months of age. CHOP-INTEND scores improved from a median score of 49 at baseline to ≥58 for all participants by 18 months (*p* < 0.0001). AEs included mild, transient hepatotoxicity (21%) and thrombocytopenia (21%). There were no treatment-related SAEs [23].

### 2.2. Post-Approval and Extension Studies

#### 2.2.1. Nusinersen

Post-approval clinical trial data for nusinersen in SMA Types 1 and 2 primarily come from extension studies following initial phase 1b/2a and phase 3 trials. One key extension study (NCT01703988 and NCT02052791) involved 28 children with later-onset SMA (11 with Type 2 and 17 with Type 3) treated with intrathecal nusinersen over approximately 3 years. By day 1150, mean motor function improvements were significant: HFMSE scores increased by +10.8 points in Type 2 SMA (*p* < 0.05), while ULM scores improved by +4.0 points (*p* < 0.05). No treatment discontinuations due to AEs were reported, and compound muscle action potential (CMAP) values remained stable, indicating sustained motor neuron function [21]. Additionally, the EMBRACE phase 2 study (NCT02462759) evaluated nusinersen in infants and children with infantile- or later-onset SMA who were ineligible for earlier pivotal trials. Over a mean 2.4-year follow-up, nusinersen showed a favorable safety profile with no related SAEs leading to discontinuation. Motor milestone responder rates were 93% in the nusinersen group vs. 29% in the sham group during the randomized phase. Ventilator support use was lower in nusinersen-treated participants, and plasma biomarkers (pNF-H) declined during loading doses, correlating with motor improvements [24].

The SHINE trial, which is a multi-center, open-label, phase 1b/2a clinical trial with an extension study by Darras et al. (2019), evaluated the long-term safety and efficacy of intrathecal nusinersen in children with later-onset SMA. Participants across multiple centers in the U.S. from the phase 1b/2a study (CS2; NCT01703988) who were eligible to enroll in the extension study (CS12; NCT02052791) were enrolled. The study population included patients with genetically confirmed 5q SMA, with a clinical diagnosis of later-onset SMA, and aged 2–15 years. In CS2, participants received an increasing dose (3–12 mg) of nusinersen over 253 days, followed by a 715-day extension in CS12, where all participants received 12.0 mg every 14 months. There were 28 participants, either with Type 2 SMA (n = 11) or Type 3 (n = 17). Data collected were demographics and clinical characteristics, with outcomes assessed using HFMSE, ULM, 6-Minute Walking Test (6MWT), CMAP, and Motor Unit Number Estimation (MUNE). At day 1150, mean HFMSE scores improved by 10.8 points among patients with Type 2. Clinically meaningful improvements, i.e., ≥3 points, were observed in 78% patients with Type 2 SMA by day 1050. ULM scores improved by 4.0 points, with 56% patients achieving clinically meaningful gains in function. One participant gained independent ambulation. CMAP and MUNE remained stable among participants with Type 2 SMA. All participants experienced at least one AE, which were mostly mild or moderate and did not lead to treatment discontinuation [25].

A multi-center prospective observational cohort study by Tachibana et al. reported the interim results from post-marketing surveillance studying the effectiveness and safety of nusinersen in patients with SMA in Japan. Patients with genetically confirmed SMA who received at least one dose of nusinersen were enrolled and treated with nusinersen between August 2017 and May 2022. A total of 522 patients, either Type 1 SMA (n = 153), Type 2 (n = 208), or Type 3 (n = 154), were included. There were improvements in motor function across all SMA types, including HINE-2 scores increasing among patients with Type 1 SMA throughout the 34-month follow-up period (*p* < 0.001) and sustained improvements in HFMSE and 6MWT scores in patients with Type 2 SMA up to 33 months (*p* < 0.001). Clinically meaningful improvements in HFMSE (≥3 points) were seen in 28.9% of patients with Type 2 SMA. Clinical Global Impressions of Improvement (CGI-I) were either improved or unchanged in 100% of the patients. AEs were reported in 35.9% of patients, including pyrexia, pneumonia, and headache. SAEs occurred in 14.1% of patients, whereas treatment-related deaths were rare [26].

#### 2.2.2. Risdiplam

For risdiplam, the available data from post-approval extension studies are limited and include ongoing trials such as MANATEE, which is evaluating the safety and efficacy in patients aged 2–25 years [27].

The JEWELFISH study by Chiriboga et al. was a multi-center, open-label, phase 2 trial that enrolled 174 pediatric and adult patients with genetically confirmed 5q-SMA who had previously received at least one dose of another DMD (nusinersen, onasemnogene abeparvovec, olesoxime, or RG7800). Participants, including those with Types 1 and 2 SMA, were recruited from 24 centers across Europe and the United States and received daily oral risdiplam at age- and weight-appropriate doses for at least 24 months. The primary objectives were to assess safety, tolerability, and PK; exploratory efficacy endpoints included changes in motor function (using MFM32, HFMSE, RULM, HINE-2, and BSID-III), respiratory function, and independence in daily living. A total of 174 patients received risdiplam, including 33 with Type 1 SMA and 86 with Type 2 SMA. The cohort was heterogeneous, with a median age of 14 years (range 1–60), and most patients (78%) had three SMN2 copies. After 24 months of treatment, the safety profile of risdiplam was favorable: 96% of patients experienced at least one AE, most commonly pyrexia (24%), headache (19%), diarrhea (17%), and nasopharyngitis (16%). The most frequent SAE was pneumonia (3%). Importantly, both AE and SAE rates decreased by more than 50% from the first to the second year of treatment, and no treatment-related AEs led to withdrawal. Only one SAE (supraventricular tachycardia) was considered related to risdiplam, occurring in a patient previously treated with olesoxime; no treatment-related SAEs were reported in patients previously treated with nusinersen or onasemnogene abeparvovec. There were no new safety signals, and no deaths related to risdiplam. Exploratory efficacy analyses showed that SMN protein levels in blood increased after risdiplam initiation and were sustained over 24 months, regardless of prior therapy. In both Type 1 and Type 2 SMA patients, mean scores on the MFM32, HFMSE, and RULM remained stable over 24 months, indicating maintenance of motor function in a population with longstanding disease and prior exposure to other DMDs. For younger children assessed with HINE-2 and BSID-III, motor function was also stable. No clinically meaningful declines in respiratory function or independence in daily living were observed in either Type 1 or Type 2 SMA patients [28].

#### 2.2.3. Onasemnogene Abeparvovec

A multi-center, open-label, phase 2 dose-escalation study by Day et al. (2021) evaluated the safety, tolerability, and efficacy of intrathecal onasemnogene abeparvovec in children with Type 2 and Type 3 SMA. Patients across five centers in the United States were enrolled. Participants received a single intrathecal dose of onasemnogene abeparvovec at either 6.0 × 10^13^ vg or 1.2 × 10^14^ vg, and were followed for 12 months. A total of 15 patients were enrolled, either Type 2 SMA (n = 3) or Type 3 (n = 12), aged 6 to <60 months. HFMSE increased from the baseline by 6.0 at day 183 and 7.7 at day 365 (no *p*-values reported); 9/15 patients achieved clinically meaningful improvements (≥3 points). No patients required ventilatory or nutritional support. AEs reported were mostly mild to moderate, including elevated transaminases resolving with corticosteroids; two (13%) patients experienced SAEs [20].

### 2.3. RWE Studies

#### 2.3.1. Nusinersen

A prospective cohort study by Aragon-Gawinska et al. (2018) evaluated the safety and effectiveness of nusinersen in patients with Type 1 SMA and who were older than 7 months. Patients were treated with intrathecal nusinersen as part of an Expanded Access Program (EAP) and assessed at baseline (M0), 2 months (M2), and 6 months (M6) post-treatment initiation. Clinical data collected included survival, respiratory and nutritional support status, and motor function assessed by the modified HINE-2, CHOP-INTEND, and MFM scales appropriate for age. Respiratory support was categorized by duration and invasiveness, and nutritional support by type. There were 33 patients with Type 1 SMA, aged 8.3 to 113.1 months. At 6 months follow-up, all 33 patients were alive and continuing nusinersen treatment. Median improvement in the modified HINE-2 motor milestone score was 1.5 points (*p* < 0.001), indicating significant motor function gains regardless of SMN2 copy number. CHOP-INTEND scores improved by a median of 4.0 points at M6, reflecting enhanced motor abilities. Five patients (16.6%) achieved stable, support-free sitting, a milestone not typically attained in untreated Type 1 SMA. Respiratory support needs increased significantly over time, with eight patients experiencing worsened respiratory status; three patients required full-time invasive ventilation, all of whom showed no motor improvement. Nutritional support remained largely stable. Hospitalizations were primarily due to respiratory complications, with no major safety concerns or laboratory abnormalities reported. The motor response was variable but notable even in older children, supporting nusinersen’s benefit beyond the previously studied younger age groups. SMN2 copy number did not significantly influence motor or respiratory outcomes [29].

A multi-center observational study by the same author group analyzed data from patients with genetically and clinically confirmed Type 1 SMA treated with nusinersen at three European centers. All patients received standard of care plus nusinersen and were enrolled in the European Registry of Patients with Infantile-onset Spinal Muscular Atrophy. Motor function was assessed using the HINE-2 and CHOP-INTEND scales at baseline, and at 2, 6, 10, and 14 months after starting treatment. Acquisition of independent sitting was defined as sitting unassisted for at least 30 s (HINE-2 score 3 or 4). The study compared sitters and non-sitters at 14 months post-treatment in relation to baseline motor function, SMN2 copy number, age at treatment initiation, and early motor improvement. Of the 53 patients initially included, 50 were genetically confirmed to have SMA, mean age of 22.0 (SD 20.7), and 56% were male; 47 completed 14 months of nusinersen treatment with known sitting status. After 14 months, 15 of 47 patients (32%) achieved the ability to sit independently, a milestone rarely seen in untreated Type 1 SMA. Five patients attained this milestone after only 6 months of therapy. Baseline characteristics, including age at symptom onset, age at treatment initiation, disease duration before treatment, and SMN2 copy number, were similar between sitters and non-sitters, with no statistically significant differences. However, sitters had significantly higher baseline HINE-2 (median 2.0 vs. 1.0; *p* < 0.01) and CHOP-INTEND scores (median 35.5 vs. 26.5; *p* < 0.05) compared to non-sitters. Furthermore, sitters demonstrated a greater median improvement in HINE-2 score at 6 months (3.0 vs. 1.0; *p* < 0.05). Patients with a baseline HINE-2 score ≥ 2 had a threefold greater likelihood of achieving independent sitting (relative risk [RR] 3; 95% CI: 1.1–8.1), and those with a gain of ≥2 points in HINE-2 at 6 months were also three times more likely to become sitters (RR 3.06; 95% CI 1.0–9.5). The combination of higher baseline function and early improvement was most predictive: patients with both a baseline HINE-2 ≥ 2 and a ≥2-point gain at 6 months had a fourfold increased probability of sitting independently (RR 4.29; 95% CI 1.8–10.4). Importantly, SMN2 copy number did not predict sitting acquisition. Two patients died during the study due to respiratory failure unrelated to treatment, and one patient discontinued therapy due to lack of motor gain and respiratory decline [30].

A retrospective, nationwide study by Belančić et al. included all Croatian patients with genetically confirmed Types 1, 2, and 3 SMA who received nusinersen reimbursed by the Croatian Health Insurance Fund (CHIF) between April 2018 and February 2022. Data were collected anonymously from the national health insurance database and reimbursement documentation, including demographics, SMA type, SMN1/SMN2 copy numbers, age at treatment initiation, and motor function scores. All patients who received at least one dose were included in baseline and safety analyses, while only those who completed six doses with available follow-up data were included in the effectiveness analysis. Motor function was assessed with CHOP-INTEND for non-sitters and young children, HFMSE for sitters and ambulant children, and Revised Hammersmith Scale (RHS) or the 6MWT for ambulant adults. A total of 52 patients, median age of 13.4 years (range 0.1–51.1) and 61.5% male, received nusinersen during the study period. At baseline, 12 patients required mechanical ventilation (10 with Type 1 SMA, two with Type 2 SMA), and 10 required nutritional support via gastrostomy or nasogastric tube. Statistically significant motor function improvement was observed in pediatric patients with Types 1 and 3 SMA. In Type 1 SMA, the mean CHOP-INTEND score improved from 10.8 ± 10.3 at baseline to 20.0 ± 15.8 after four loading doses (*p* = 0.003), with the effect persisting through subsequent doses. Notably, both ventilated and non-ventilated patients with Type 1 SMA showed improvement, though gains were greater in those not requiring ventilation. In Type 2 SMA, average HFMSE scores increased by 6.0, 10.5, and 11.0 points after four, five, and six doses, respectively. Across all groups, earlier initiation of nusinersen was associated with greater motor gains (negative correlation between age at treatment and motor improvement, rS = −0.77, *p* = 0.001). During the study, 437 doses were administered without any new safety concerns; AEs were mild and related to lumbar puncture. No patients discontinued treatment due to AEs [31].

A retrospective, observational cohort study by Chan et al. (2021) included patients with genetically confirmed Type 1 SMA from eight centers in Hong Kong, Taiwan, and South Korea who began nusinersen treatment under an EAP between 2017 and 2019. Baseline demographic and clinical data—including SMN1 mutation, SMN2 copy number, age at symptom onset, age at treatment initiation, and respiratory and feeding status—were collected. Motor function was assessed at baseline, 6 months, and 10 months after treatment initiation using the HINE-2 and CHOP-INTEND scales. Clinically meaningful improvement was defined as a CHOP-INTEND increase of ≥4 points or a HINE-2 gain of ≥5 points. Forty patients with Type 1 SMA were included, with a median age at nusinersen initiation of 20 months (range 0.35–294 months); two-thirds had two SMN2 copies, and over half started treatment at ≤2 years of age. Nine patients were identified by newborn screening, most of whom began nusinersen before 7 months of age. All patients started nusinersen after symptom onset. At one year, 95% of patients remained in the program; one died of respiratory failure before 6 months, and one discontinued due to lack of improvement, both with two SMN2 copies. Motor outcomes were significantly better in patients who started nusinersen at ≤2 years of age: 36.4% achieved unassisted sitting and 13.6% attained assisted standing by 10 months, with 61.1% gaining ≥5 HINE-2 points (median gain 7.5). In contrast, among those starting treatment after 2 years, only 6.7% achieved unassisted sitting and 7.1% gained ≥5 HINE-2 points (median gain 0.5). Patients with three SMN2 copies had greater improvements in HINE-2 and CHOP-INTEND scores compared to those with two copies (*p* = 0.003 and *p* < 0.001, respectively). Early treatment initiation, shorter disease duration, higher baseline HINE-2 scores, and identification by newborn screening were associated with greater motor gains. Multiple regression revealed that newborn screening was the only independent predictor of HINE-2 improvement at 10 months [32].

A single-center observational cohort study by Chen et al. (2020) included children aged 0–18 years with genetically confirmed Type 1 SMA who began nusinersen treatment at Sydney Children’s Hospital Network between November 2016 and September 2018, with follow-up to October 2019. Patients were classified as newly diagnosed or chronic based on the timing of treatment initiation. Nusinersen was administered intrathecally according to standard dosing protocols. Primary outcomes included the need for respiratory support (non-invasive ventilation [NIV]), bulbar dysfunction (assessed by swallow studies and clinical evaluation), nutritional support (nasogastric or gastrostomy feeding), and hospitalization burden. Motor function was assessed using the CHOP-INTEND scale. Nine children (five females, four males; median age at treatment initiation 10.7 months, range 2.7–181.2) were included and followed for a mean of 30.1 months, accounting for 270.5 patient-months and 209 hospital admissions. All patients survived throughout the study period. Among newly diagnosed patients (n = 7), four required gastrostomy insertion and four commenced NIV, typically within the first year after diagnosis. Children with two SMN2 copies had a significantly higher need for gastrostomy and more frequent hospital admissions compared to those with three SMN2 copies (*p* < 0.05). Bulbar dysfunction was common: three of five newly diagnosed patients who underwent swallow studies had evidence of aspiration and required percutaneous endoscopic gastrostomy (PEG) feeding, all with two SMN2 copies. Management of bulbar dysfunction included pharmacological therapy for secretion control in two patients. The annualized hospitalization rate was 9.3 per patient per year, with an average length of stay of 3.3 days. Notably, the number of hospital admissions halved from the first to the second year of nusinersen treatment (*p* < 0.005), reflecting a reduction in unplanned acute illness admissions over time. AEs related to nusinersen administration were mild, including contact dermatitis and headache; no patient discontinued treatment. Motor function, assessed by CHOP-INTEND, showed clinically meaningful improvement in several patients, but all continued to require substantial supportive care [33].

A multi-center, longitudinal registry study in China by Yao et al. (2024) included pediatric patients under 18 years with genetically confirmed 5q-SMA who initiated nusinersen treatment from April 2019 onward. Data were collected both retrospectively and prospectively from 18 centers, with motor function assessments planned at baseline and approximately 6, 10, and 14 months after treatment initiation. Motor outcomes were evaluated using standardized scales appropriate for SMA subtype: CHOP-INTEND and HINE-2 for Type 1 patients, and HFMSE and RULM for Type 2 patients. Safety data were collected prospectively for patients who started nusinersen after registry enrolment. Among 385 pediatric patients, 41 had Type 1 SMA and 214 had Type 2 SMA. The median age at nusinersen initiation was 42 months for Type 1 and 62.5 months for Type 2 patients. At 6 and 10 months post-treatment, patients with Type 1 SMA demonstrated significant improvements in CHOP-INTEND scores, with many showing motor gains consistent with improved neuromuscular function. For patients with Type 2 SMA, mean changes in HFMSE scores were 4.4 (95% CI: 3.4–5.4) at 6 months and 4.1 (95% CI: 2.8–5.4) at 10 months, indicating meaningful motor function improvement. Similarly, RULM scores improved by 2.4 (95% CI: 1.7–3.1) and 2.3 (95% CI: 1.2–3.4) at these time points, reflecting enhanced upper limb function. Most patients across both types showed either motor improvement or stabilization. Safety analysis of 132 patients initiating nusinersen post-enrolment revealed that 62.9% experienced AEs, predominantly mild and unrelated to treatment; only two patients had mild AEs considered related to nusinersen (aseptic meningitis and myalgia), with no lasting sequelae [34].

A prospective, multi-center, post-marketing surveillance study by Jiang et al. (PANDA, NCT04419233) evaluated the safety, efficacy, and PK of nusinersen in children with genetically confirmed 5q-SMA in routine clinical practice across China. Participants were enrolled consecutively and observed for two years following nusinersen initiation, with dosing according to the approved regimen (four loading doses followed by maintenance every four months). The primary endpoint was the incidence of AEs and SAEs during the treatment period. Efficacy assessments included WHO motor milestones, HINE, and the need for ventilatory support, with evaluations conducted at regular intervals. Plasma and cerebrospinal fluid (CSF) concentrations of nusinersen were measured at each dose visit for PK analysis. Data were analyzed descriptively, with subgroup analyses by age of symptom onset (≤6 months [infantile-onset, generally Type 1 SMA] and >6 months [later-onset, generally Type 2 SMA]). As of the 4 January 2023 data cutoff, 50 pediatric participants were enrolled: 10 with infantile-onset SMA (onset ≤ 6 months, corresponding to Type 1 SMA) and 40 with later-onset SMA (onset > 6 months, corresponding predominantly to Type 2 SMA). All participants received at least one dose of nusinersen, and six had completed the study at the time of interim analysis. AEs were reported in 45 participants (90%), with the majority being mild or moderate in severity; no AEs led to discontinuation of nusinersen or study withdrawal. Eleven participants experienced SAEs, most commonly pneumonia (n = 9), but none were considered related to nusinersen treatment. Importantly, no serious respiratory events occurred, and no participant required initiation of permanent ventilatory support during the study period. Regarding efficacy, both infantile-onset (Type 1) and later-onset (Type 2) SMA subgroups demonstrated stability or gains in WHO motor milestones throughout the study. Mean HINE-2 scores improved in both subgroups, indicating clinically meaningful motor function gains. In the infantile-onset group, some patients achieved new motor milestones, such as improved head control or independent sitting, while in the later-onset group, maintenance or improvement of motor abilities was observed. No deaths were reported, and the overall safety profile was consistent with previous clinical experience with nusinersen. PK analysis showed that pre-dose CSF concentrations of nusinersen increased steadily through the loading-dose period, with no evidence of plasma accumulation after multiple doses [35].

A retrospective, multi-center study by Wang et al. evaluated adolescent and adult patients with genetically confirmed 5q-SMA who received nusinersen at three neuromuscular centers in China between October 2022 and July 2023. Eligible patients were aged 13 years or older and had completed at least four loading doses of nusinersen, followed by maintenance dosing according to standard protocols. Patients were categorized as “sitters” or “walkers” based on their baseline motor function. Motor outcomes were assessed at baseline, day 63, day 180, and day 300 using the HFMSE, RULM, 6MWT, and percent-predicted forced vital capacity (FVC%). Electrophysiological assessments included CMAP amplitude measurements in selected upper and lower limb muscles, performed at baseline and at least once after treatment. Of the 54 patients included, 9 had Type 2 SMA and none had Type 1 SMA, with the remainder having Type 3 or 4. The median age at therapy initiation for Type 2 patients was lower, and all Type 2 patients were classified as “sitters.” Baseline HFMSE scores for Type 2 patients averaged 13.5, reflecting significant motor impairment. After nusinersen treatment, sitters—including all Type 2 patients—showed a mean HFMSE increase of 1.12 points at day 63, 2.32 points at day 180, and 3.21 points at day 300, with the change at day 63 reaching statistical significance (*p* = 0.040) and a positive trend at subsequent time points. RULM scores in sitters did not change significantly, although a positive trend was observed, and 11–26% of all patients achieved a clinically meaningful improvement (≥2 points) across time points. FVC% did not significantly improve in sitters, but a positive trend was noted. Electrophysiological analysis in a subset of patients, including sitters, demonstrated significant increases in CMAP amplitudes in both upper and lower limbs after treatment, suggesting improved neuromuscular transmission. Nusinersen was well tolerated, with the most common AEs being post-lumbar puncture headache and lumbar pain; no SAEs attributable to nusinersen were reported [36].

A retrospective, multi-center study by Li et al. was conducted across three neuromuscular centers in China from October 2022 to July 2023 to evaluate the effectiveness and safety of nusinersen in adolescent and adult patients with genetically confirmed 5q-SMA. Eligible participants were aged 13 years or older and had a confirmed diagnosis of SMA based on homozygous deletion of exon 7 or compound heterozygosity for pathogenic mutations in the SMN1 gene. Patients received the standard nusinersen dosing regimen, consisting of four intrathecal loading doses (12 mg each) administered at baseline, day 14, day 28, and day 63, followed by maintenance doses every four months. Motor function was assessed at baseline and at days 63, 180, and 300 using the HFMSE, RULM, 6MWT, and FVC%. Electrophysiological assessments included CMAP amplitude measurements in selected upper and lower limb muscles, performed at baseline and at least once after treatment. Patients were categorized into functional subgroups based on their motor abilities, including sitters and walkers. Treatment response was defined by clinically meaningful improvements in motor scales or walking distance. Safety was monitored through clinical evaluation and laboratory tests at each visit. Statistical analyses included paired comparisons over time, correlation analyses, and logistic regression to identify predictors of treatment response. In the study cohort, nine patients with Type 2 SMA were included; however, data for Type 1 SMA patients were either not explicitly reported or the number was too small for separate analysis. Among the Type 2 subgroup, motor function outcomes showed modest improvements following nusinersen treatment. Although detailed numeric values for Type 2 patients alone were not separately tabulated, the overall cohort—including Type 2 patients—demonstrated trends toward motor function improvement as measured by HFMSE and RULM. Specifically, the mean baseline HFMSE score for the entire cohort was 32.61 ± 15.25, and while subgroup-specific scores were not provided, the Type 2 patients likely contributed to the lower range of baseline function given their disease severity. Electrophysiological assessments revealed increases in CMAP amplitudes in both upper and lower limb muscles after nusinersen treatment. Although the paper did not provide separate CMAP amplitude data for Type 2 patients, the overall improvements in CMAP amplitudes suggest enhanced neuromuscular function that may also apply to this subgroup. Regarding safety, nusinersen was well tolerated in all patients, including those with Type 2 SMA. The most commonly reported AEs were post-lumbar puncture headache and lumbar pain, which were transient and manageable. No SAEs related to nusinersen were reported in the Type 2 subgroup or the overall cohort. Laboratory parameters, including liver enzymes and creatine kinase, remained stable throughout the study [37].

A prospective observational study by Gaboli et al. was conducted at Hospital Universitario Virgen del Rocío in Spain and enrolled pediatric patients with genetically confirmed Types 1–3 SMA who received nusinersen for at least 24 months. All patients had SMN1 gene deletions or mutations and were included consecutively without exclusion criteria. Clinical and genetic data, including SMA type, SMN2 copy number, age at diagnosis, age at nusinersen initiation, and respiratory and bulbar status, were collected. Functional status was assessed using WHO motor milestones (sitting and walking), and respiratory function was evaluated by spirometry (FVC and FEV1 z-scores) in patients over six years old. The need for mechanical ventilation and feeding support was also documented. The study enrolled 28 children: 11 with Type 1 SMA (6 Type 1b, 5 Type 1c) and 12 with Type 2 SMA. At baseline, all Type 1 patients were unable to sit independently, while all Type 2 patients could sit, and some could walk. Patients with Type 1 SMA were diagnosed and treated significantly earlier than those with Type 2 SMA (*p* < 0.001), and most patients with Type 1 (81.8%) had two SMN2 copies, compared to 11.8% in Types 2 and 3. Over 24 months of nusinersen treatment, functional gains were observed: by 6 months, one Type 1b and three Type 1c patients achieved independent sitting, and by 24 months, three Type 1b patients could sit independently. In Type 2 patients, two achieved independent walking at 6 months, and three more showed further functional improvement by 12 months. Earlier initiation of nusinersen was associated with greater odds of functional improvement at each follow-up (odds ratio 2.30 at 6 months, 3.93 at 12 months, and 4.64 at 24 months; all *p* < 0.05). In Type 2 SMA, pulmonary function improved significantly: mean FVC z-score increased from −3.55 at baseline to −1.07 at 6 months (*p* = 0.002), −1.99 at 12 months (*p* = 0.018), and −1.69 at 24 months (*p* = 0.013); mean FEV1 z-score improved from −3.52 at baseline to −1.62 at 6 months (*p* = 0.011), −2.04 at 12 months (*p* = 0.035), and −1.81 at 24 months (*p* = 0.020). The need for mechanical ventilation remained higher in patients with Type 1 than Type 2, with 63.6% of Type 1 and 25% of Type 2 patients requiring some form of ventilation at baseline, and a proportion of Type 1 patients needing permanent invasive support. Bulbar function, including swallowing and feeding, remained stable, with all Type 1c patients maintaining oral feeding, while Type 1b patients were more likely to require alternative feeding support. No deaths or unexpected safety signals occurred during the study [38].

A single-center, retrospective observational cohort study in Romania by Axente et al. (2022) evaluated the clinical and electrophysiological outcomes in pediatric patients with Types 1, 2, and 3 SMA treated with nusinersen for 26 months. A total of 34 patients with genetically confirmed SMA were enrolled, among whom 33 have SMN1 deletions and one is a compound heterozygous case, with ages ranging from 1 to 16 years, carrying 2–3 SMN2 copies. Motor function was assessed at baseline at every 4 h post-initiation of nusinersen using CHOP-INTEND for non-sitters (Type 1 SMA), HFMSE for sitters and walkers (Type 2/3 SMA), and 6MWT for ambulatory patients, and also distal CMAP readings for the ulnar nerve. Overall, 45% of Type 1 SMA non-sitters were able to sit, and 25% of Type 2 SMA sitters were able to walk after 10 doses of nusinersen. Improvements in motor function were significantly associated with increases in CMAP amplitude among Type 1 SMA patients (r = 0.667; *p* < 0.005), but not among Types 2 or 3 SMA, where outcomes remained stagnant. Better outcomes were associated with earlier treatment initiation and higher baseline CMAP altitudes [39].

A single-center, prospective, observational cohort study by Chacko et al. (2022) evaluated the effectiveness of nusinersen on respiratory function among children with Types 1–3 SMA during the first year of treatment in Queensland, Australia. Only patients under 19 years old were included, who did not previously take nusinersen, have a genetically confirmed homozygous SMN1 mutation, with features that are consistent with Types 1, 2, or 3 SMA. Data were collected through respiratory and motor function measures that are appropriate for the patient’s age, including spirometry, oscillometry, sniff nasal inspiratory pressure (SNIP), mean inspiratory/expiratory pressures, lung clearance index (LCI), and polysomnography (PSG); baseline and post-treatment at one and two years lung function data were compared. Motor function was also tested using CHOP-INTEND, HFMSE, and RULM. There were 28 patients, aged 0.08–18.58 years, among whom 15 were males, with genetically confirmed SMA and with clinical features of SMA Types 1 (n = 7), 2 (n = 12), or 3 (n = 9). The yearly decline rate of FVC z-score slowed post-treatment, particularly among Type 2 SMA (*p* = 0.002). The total apnea–hypopnea index significantly decreased among Type 1 SMA patients (median AHI 5.5–2.7 events/hour; *p* = 0.02). There were improvements in the peripheral motor functions among all (100%) Type 1 SMA patients, and 75% among those with Types 2 and 3 SMA. There were no new safety signals reported, though there was one reported death due to respiratory complications in a Type 1 SMA patient [40].

A multi-center, longitudinal observational cohort study by Cho et al. (2023) examined the effectiveness and safety of nusinersen among Types 1, 2, and 3 SMA, using real-world data from South Korea. Using the Korean Health Insurance Review and Assessment Service database, patients who were treated under the national health insurance reimbursement scheme were identified. Investigators only included patients with genetically confirmed 5q-SMA, whose symptoms appeared before 3 years old, with no permanent respiratory support, with available motor function data, who were treated with nusinersen for at least 6 months. Motor function was assessed using HINE-2 for Type 1 SMA and HFMSE for Types 2 and 3 SMA, collected at baseline and at certain intervals until 3 years. A total of 137 patients were included, 71 were males, either SMA Types 1 (n = 21), 2 (n = 103), or 3 (n = 13). Among those with Type 1 SMA, the mean HINE-2 scores increased by +6.6 points at year 1, +3.9 points at year 2, and +0.8 points at year 3. Earlier treatment, i.e., <18 months of symptom onset, was associated with significantly higher increases in HINE-2 scores, compared to later initiation (*p* = 0.02 at year 2; *p* = 0.03 at 30-month follow-up). Among those with Type 2 SMA, the mean HFMSE increased by +4.7 at year 1, +6.9 at year 2, and +9.1 at year 3. Likewise, patients who were treated earlier had significantly better improvement at year 2 compared to those who were treated later (+8.4 vs. +6.8; *p* = 0.001). Subgroup analysis revealed that motor function gains were the lowest among patients with the lowest baseline function, i.e., HFMSE = 0, though 60% of these patients still achieved improvements that were clinically meaningful. There were no SAEs requiring withdrawal from treatment [41].

A two-center prospective observational cohort study by Osredkar et al. (2021) was conducted to evaluate the effectiveness and safety of nusinersen in children and adolescents with SMA over a 14-month follow-up between March 2017 and November 2018 in Slovenia and the Czech Republic. A total of 61 patients were included, either SMA Type 1 (n = 16), 2 (n = 32), or 3 (n = 13), all under 19 years of age. Significant improvements in motor function scores among those with Type 1 SMA (*p* < 0.002) and Type 2 SMA (*p* < 0.002) were reported; there was an increasing trend among those with Type 3 SMA, but it was not significant (*p* = 0.051). Younger age at treatment initiation (*p* = 0.016), longer treatment duration (*p* < 0.001), and a higher SMN2 copy number (*p* = 0.020) were independently associated with better motor outcomes. At 14 months follow-up, 72.9% patients had improvement in their motor function, whereas 11.9% remained stable. More importantly, there were five Type 1 SMA patients who gained the ability to sit and five Type 2 SMA patients who gained the ability to stand or walk. No SAEs were reported, but 39.3% patients experienced minor AEs, including lumbar pain or headache, primarily during the loading phase [42].

A single-center, retrospective observational cohort study by Mirea et al. (2022) was conducted to examine whether physical therapy (PT) enhanced motor function in children and adolescents with SMA receiving nusinersen therapy at the National Teaching Center for Children’s Neurorehabilitation in Romania between October 2018 and June 2021. Patients with genetically confirmed SMA Types 1, 2, or 3, with deletion of SMN1 exon 7 for both the control (N) and intervention group (PT-N), were enrolled, with the latter having had at least five sessions of PT per week. Patients who had PT sessions less than once a week or had undergone spinal surgery in the control group (N) were included. Motor function data were collected at baseline, 6, and 12 months, using the CHOP-INTEND scale for Type 1 SMA and HGMSE for Types 2 and 3 SMA. Overall, 55 patients were included, either SMA Type 1 (n = 20), 2 (n = 26), or 3 (n = 9). At 12 months follow-up, patients in the PT-N group showed significantly increased motor function scores compared to those in the N group (mean increase of 23.9% vs. 5.4%; *p* < 0.001), with Type 1 SMA obtaining the highest gains. CHOP-INTEND scores increased with a mean of 33.2% in the PT-N group with Type 1 SMA, compared to 6.9% in the N group [43].

A multi-center, prospective observational cohort study by Modrzejewska et al. (2021) examined the effectiveness and safety of nusinersen in children with Type 1 SMA in the EAP in Poland. Patients from 3 EAP centers were enrolled between February 2017 and March 2019. Investigators included patients with genetically confirmed Type 1 SMA with biallelic deletion of the SMN1 gene, confirmation of SMN2 copy number, and without contraindications for lumbar puncture. Patients received 12.0 mg of intrathecal nusinersen following a standard dosing regimen, with a follow-up during the administration of the 8th to 10th dose, or at 18–26 months. A total of 26 Type 1 SMA patients were enrolled, 4.79 years (range 2–15 years), and 14 were males. The CHOP-INTEND scores improved from 19.1 ± 14.3 at baseline to 26.5± 18.0 at follow-up (mean +7.4 points, 95% CI: 4.7–10.1; *p* < 0.001). Those with three or more copies of SMN2 had significantly higher CHOP-INTEND scores (*p* = 0.013), but did not show significant improvement over time (*p* = 0.324). There was a more significant improvement among those with baseline scores above the cohort median (*p* = 0.037). All patients improved or stabilized in terms of ventilatory and nutrition support. The treatment was also well tolerated, without SAEs. Mild, transient events included post-lumbar puncture syndrome (15.4%), respiratory infections (15.4%), elevated liver enzymes (7.7%), and temporary CSF leakage (7.7%) [44].

A single-center, prospective, observational cohort study by Olsson et al. (2019) studied the CSF biomarkers as indicators of response to nusinersen among children with Type 1 SMA. Children with genetically confirmed Type 1 SMA with two SMN2 gene copies who received nusinersen at Queen Silvia Children’s Hospital, Sweden, were enrolled. CSF samples were collected at baseline and during each visit for intrathecal administration of nisunersen within a window of around 11 months, and levels of neurofilament light (NFL), tau, and glial fibrillary acidic protein (GFAP) were measured using ELISA. A total of 12 Type 1 SMA patients and 11 age-matched controls were included. Initially at baseline, Type 1 SMA patients showed significantly high levels of NFL (4598 ± 981 pg/mL vs. 148 ± 39 pg/mL; *p* = 0.001), tau (939 ± 159 pg/mL vs. 404 ± 86 pg/mL; *p* = 0.02), and GFAP (236 ± 44 pg/mL vs. 108 ± 26 pg/mL; *p* = 0.02) compared to controls. With treatment, NFL levels decrease significantly (−879.5 pg/mL per dose, 95% CI: −1343.4–−415.6; *p* = 0.0001), reaching normal levels at the fourth or fifth dose. Likewise, tau and GFAP declined (−112.6 pg/mL/dose; *p* = 0.01; −16.9 pg/mL/dose; *p* = 0.02, respectively). There were improvements in motor function in all children receiving more than one dose, with an increase in median CHOP-INTEND scores by 13 points (5.4 points/month; <0.0.0001). Improvements in scores were significantly correlated with reductions in NFL (rho = −0.64; *p* = 0.03) and tau (rho = −0.85; *p* = 0.0008), but not GFAP [45].

A multi-center, prospective observational cohort study by Pane et al. (2023) studied the effectiveness and safety of nusinersen among children with Type 1 SMA over a 4-year follow-up period. Patients with Type 1 SMA who received nusinersen through the early access program in Italy were included. A total of 48 patients, mean age at treatment was 3.3 ± 3.6 years (ranging from 7 days to 12 years), who had at least one assessment at 12, 24, and 48 months in treatment, were enrolled. Over 48 months, CHOP-INTEND scores improved (significantly mean change: 10.6 ± 12.1; *p* < 0.001), with 77.1% of patients demonstrating a ≥4-point gain, and HINE-2 scores improved (mean change: 4.3 ± 5.7; *p* < 0.001). Improvements were highest among those who initiated treatment before 2 years old (*p* < 0.001) and among those with milder baseline severity of their disease. Respiratory and nutritional function remained stable, if not improved over time. Only one patient died during the follow-up period. There were no treatment-related SAEs. Mild AEs, such as headache and nausea, occurred only in 20.8% of patients [46].

A multi-center prospective observational cohort study by Pechmann et al. (2018) was conducted to evaluate the short-term effectiveness and safety of nusinersen in children with Type 1 SMA treated within Germany’s EAP in seven neuromuscular centers between November 2016 and June 2017. Only children with genetically confirmed 5q-SMA with symptom onset before 6 months of age and inability to sit independently were included. Data on motor function using CHOP-INTEND and HINE-2 at baseline and at 60 and 180 days post-treatment initiation were collected. A total of 61 patients with Type 1 SMA were enrolled, with a mean age of treatment initiation at 21.1 months (range 1–93). At 180 days, 77% of the children had an improvement of ≥4 points on CHOP-INTEND scores (mean change: 9.0 ± 8.0); 34.4% had improvement on HINE-2 scores, with 6.6% achieving full head control, and 3.3% were able to sit independently. Significant predictors of improvement on CHOP-INTEND scores included age at treatment initiation (*p* = 0.0006); particularly, children aged ≤7 months gained a mean of 14.4 ± 9.2 points increase. Respiratory and nutritional requirements remained stable, but 6.6% patients had reduced ventilatory dependence. There were no severe procedural complications; however, 54.7% experienced SAEs, including respiratory infections (58.5%) and acute respiratory failure (15.1%) [47].

A multi-center retrospective observational cohort study by Szabó et al. (2020) examined the effectiveness and safety of nusinersen among pediatric patients with Type 1, 2, or 3 SMA in Hungary. Patients under 18 years old with genetically confirmed SMA who initiated nusinersen treatment between April 2018 and December 2019 at two national treatment centers were included. Motor function was assessed using CHOP-INTEND for non-sitters and those < 2 years, HFMSE, RULM, and 6MWT, evaluating at baseline and every four months after receiving the fourth injection. A total of 54 patients were enrolled, 34 were male, either SMA Type 1 (n = 10), 2 (n = 21), or 3 (n = 23). Among those who completed six doses and were evaluated, patients with Type 1 SMA (n = 7) showed a mean improvement in CHOP-INTEND scores of 14.9 ± 5.1 points by day 307 (*p* = 0.016), and had improvement of at least 4 points; evaluated patients with Type 2 SMA (n = 16) had a mean increase in HFMSE scores of 7.2 ± 5.0 (*p* < 0.001) and a mean increase in 4.3 points in RULM scores (*p* = 0.031). Lastly, evaluated patients with Type 3 SMA (n = 15), HFMSE improved by 5.3 ± 4.4 points (*p* = 0.001), and 6MWT increased by 33.9 m (*p* = 0.007). Nusinersen was associated with favorable safety outcomes, with no treatment discontinuation noted due to AEs. Among 340 injections, there were only mild AEs, including headache (8%), backache (6%), vomiting (6%), and a single self-limiting case of thrombocytopenia [48].

A multi-center retrospective observational cohort study by Tscherter et al. (2022) examined the effectiveness and safety of nusinersen among patients in Switzerland. Data from the Swiss Registry for Neuromuscular Disorders were collected to evaluate changes in motor function, ventilation, nutrition, and language development. Patients with genetically confirmed SMA who had received nusinersen for at least six months as of August 31, 2020, were enrolled. Motor function was evaluated using CHOP-INTEND, HINE-2, HFMSE, RULM, or 6MWT. A total of 44 patients were enrolled, 21 were male, either SMA Type 1 (n = 11), 2 (n = 21), or 3 (n = 12), aged 0.1–44.6 years at treatment initiation, and a median treatment duration of 1.9 years (range 0.5–3.4). All patients with Type 1 SMA1 (n = 11) had an improvement in CHOP-INTEND scores, with a median increase of 25 points (range of 2–43 points), with higher gains among those treated before 18 months (rₛ = −0.85; 95% CI: −0.96–−0.48; *p* = 0.002). Patients with Type 2 SMA (n = 21) had improvements on HFMSE (range of 1–15 points), while motor improvement was moderately correlated with the number of SMN2 copies (rₛ = 0.55; *p* = 0.032). Around 34% of patients experienced mild, self-limiting AEs, including proteinuria, thrombocytosis, and lumbar puncture-related issues [49].

A single-center retrospective observational cohort study by Weststrate et al. was conducted to evaluate bulbar function evolution in infants with Type 1 SMA treated with nusinersen at Great Ormond Street Hospital, London. Patients with genetically confirmed Type 1 SMA and a minimum of 24 months of nusinersen treatment were included. Swallowing and feeding outcomes were assessed using the Paediatric Functional Oral Intake Scale (p-FOIS), motor function assessments using CHOP-INTEND, all at baseline, and 6, 12, and 24 months post-treatment initiation, along with nutritional status and NIV requirements. A total of 24 patients with Type 1 SMA, subdivided into Type 1a (n = 3), 1b (n = 9), and 1c (n = 12), 10 males, and a median age of 11 months (range of 1 month–7.5 years), were enrolled. There were improvements in CHOP-INTEND scores from a median of 32.0 at baseline to 42.0 at 12 and 24 months. The bulbar function outcomes, however, were less favorable. Median p-FOIS scores declined from 3.0 at baseline to 2.0 at 12 and 24 months, while the percentage of patients requiring tube-feeding increased, from 58% at baseline to 83%. Only four patients, all 1c subtype, remained fully orally fed at 24 months. Videofluoroscopy assessments among eight patients showed aspiration risks across subtypes. There was disassociation between motor improvement and persistent bulbar impairment, though no statistical tests were performed to confirm this [50].

A prospective, multi-center observational cohort study by Günther et al. (2024) was conducted to examine the effectiveness and safety of nusinersen in adults with SMA in Austria, Germany, and Switzerland. Patients with genetically confirmed SMA, aged 16–71, and who were treated continuously with nusinersen between July 2017 and May 2022 were included, with a follow-up of at least 14 months. Motor function at baseline and at 14, 26, and 38 months follow-up was assessed using the HFMSE, RULM, and 6MWT. A total of 237 patients, 135 were males, either SMA Type 1 (n = 5), 2 (n = 67), 3 (n = 156), or 4 (n = 9). HFMSE scores significantly improved over time, with a mean increase of 1.72 (95% CI 1.19–2.25; *p* < 0.0001) at 14 months, 1.20 (95% CI 0.48–1.91; *p* = 0.0012) at 26 months, and 1.52 (95% CI 0.74–2.30; *p* = 0.0002) at 38 months. Likewise, RULM significantly improved over time, with a mean increase of 0.75 (95% CI 0.43–1.07; *p* < 0.0001) at 14 months, 0.65 (95% CI 0.27–1.03; *p* = 0.0007) at 26 months, and 0.72 (95% CI 0.25–1.18; *p* = 0.0030; *p* = 0.0025) at 38 months. Among ambulatory patients, 6MWT likewise increased over time, with significant mean increases of 30.86 m (95% CI 18.34–43.38; *p* < 0.0001) at 14 months, 29.26 m (95% CI 14.87–43.65; *p* = 0.0002) at 26 months, and 32.20 m at 38 months (95% CI 10.32–54.09; *p* = 0.0048). On average, clinically meaningful improvements were observed in around 30% of the patients. Among 389 patients with safety data, 91% experienced at least one AE, which were mostly mild, self-limiting, and procedure-related, including post-lumbar puncture syndrome and headache. No new safety signals were identified [51].

A prospective multi-center observational cohort study by Hully et al. (2020) was conducted to study the palliative care practices for infants with Type 1 SMA in France, focusing on caregiver-reported outcomes and the ethics surrounding emerging treatments, particularly nusinersen. A total of 37 patients (17 were male) from 17 pediatric neuromuscular centers were included. Data were collected using a structured health book completed by both the parents and healthcare providers, which captured data on motor function and therapy, nutritional and respiratory management, pain control, and palliative care. Further, retrospective cohort data were collected from 43 patients with Type 1 SMA, including seven who received nusinersen. Compared to those who did not receive treatment, those treated were more likely to receive supportive care, including gastrostomy (43% vs. 3%; *p* = 0.004), home NIV (57% vs. 1%; *p* = 0.0058), and fewer received sedative medications at end-of-life (14% vs. 76%; *p* = 0.002). Statistically significant differences in survival were not observed (*p* = 0.06), but 4/7 patients among those who were treated with nusinersen remained alive during the study period, whereas the rest of the 3/7 receiving treatment and the rest of those not receiving treatment died [52].

A multi-center prospective observational cohort study by Łusakowska et al. (2023) assessed the effectiveness and safety of nusinersen in older children and adults with SMA, treated at two specialized centers under the Polish national reimbursement program between April 2019 and June 2021. Motor function was evaluated using HFMSE, CHOP-INTEND, RULM, and 6MWT, alongside patient-reported outcomes using the Patient Global Impression of Improvement (PGI-I) scale. A total of 120 patients were included, either SMA Type 1 (n = 12), 2 (n = 19), or 3 (n = 89), with a mean age of 32 years (range 5–66). There were significant improvements in the mean HFMSE scores from baseline to 30-month follow-up by 5.1 points (95% CI: 3.4–6.9; *p* < 0.001); 71% achieved clinically meaningful improvement (≥3 points). Likewise, there were significant improvements in CHOP-INTEND scores from baseline to 26 months by 5.6 points (*p* < 0.001); 80% reached a ≥4-point gain by 30 months. Further, there were significant improvements in RULM scorers from baseline to 30-month follow-up with a mean increase of 1.96 points (*p* < 0.001); 43.5% achieved the ≥2-point threshold. Among 27 patients, 50% had a ≥30 m improvement in 6MWT at 30 months, though results were only significantly different in earlier time points, particularly at 10-month follow-up (+22.1 m; *p* = 0.007). Overall, 85% patients reported improvements on the PGI-I scale at 30 months. AEs were mostly mild, including post-lumbar puncture syndrome [53].

A multi-center retrospective observational cohort study by Yang et al. (2023) assessed the effectiveness of nusinersen on motor function and nutritional status among pediatric patients with SMA in Western China. Patients with genetically confirmed SMA, who received four loading doses of nusinersen within the first two months, were enrolled. Motor function was evaluated using CHOP-INTEND, HINE-2, RULM, HFMSE, 6MWT, and WHO motor milestones, depending on SMA type and age. Nutritional status was evaluated using weight-for-age z-scores. Healthcare-provided AEs were documented during follow-up interviews and clinical assessments. A total of 46 patients were enrolled, either SMA Type 1 (n = 8), 2 (n = 31), or 3 (n = 7). RULM scores for Type 2 SMA increased by 1.8 over time (*p* = 0.004). Further, HFMSE significantly increased by 2.5 over time (*p* < 0.0001). Weight-for-age z-score improved significantly among patients with Type 2 SMA (*p* = 0.008). For Type 3 SMA, HFMSE increased by 3.0, RULM increased by 3.4, and 6MWT increased by 26.4, though no *p*-values were reported. For Type 1 SMA, there were increases in CHOP-INTEND by 2.5 and in HINE-2 by 0.6, but both are not significant (*p* > 0.05). AEs were mostly mild and self-limiting, including upper respiratory infections, vomiting, and post-lumbar puncture symptoms; there were no serious complications reported [54].

A multi-center prospective observational cohort study by Kotulska et al. (2022) examined the effectiveness and safety of nusinersen in pediatric patients with SMA in Poland. Data on demographics, SMA type, count of SMN2 copies, motor function scores, either CHOP-INTEND or HFMSE, were collected at baseline and at follow-up visits every quarter for at least 1 year. A total of 292 patients, either SMA Type 1 (n = 127), 2 (n = 68), or 3 (n = 93); mean age was 6.9 years. CHOP-INTEND scores improved significantly by a mean score of 8.9 points at one-year follow-up (*p* < 0.001); 75.9% of patients achieved clinically meaningful gains in CHOP-INTEND. Likewise, HFMSE scores improved by a mean score of 6.1 points (*p* < 0.001); 72.7% of patients achieved clinically meaningful gains in HFMSE. Further analysis showed that younger age and higher baseline motor function were predictors of better treatment outcomes. Further, patients with Type 2 SMA (*p* = 0.004) responded better in terms of CHOP-INTEND scores, compared to patients with Type 1 SMA. AEs were not frequent, and they were mostly mild and self-limiting, while no SAEs were reported. There were two reported deaths, but they were assessed as unrelated to the treatment [55].

A multi-center prospective observational cohort study by Gómez-García de la Banda et al. (2020) was conducted to evaluate the effectiveness of nusinersen among children with Type 1c SMA and Type 2 SMA, by comparing it with age-matched historical controls, in France. Patients with genetically confirmed Type 1c or Type 2 SMA who received six doses of nusinersen were enrolled. A total of 16 patients were enrolled, either SMA Type 1 (n = 2) or 2 (n = 14), with a mean age of 9.4 ± 2.3 years (range 7.1–11.7 years). Children treated with nusinersen had significantly greater inspiratory muscle strength compared to historical controls; particularly, the former had improvements in sniff esophageal pressure (Sniff Pes: *p* = 0.018), maximal inspiratory pressure (*p* = 0.003), and FVC (*p* = 0.029). Likewise, HINE-2 (*p* < 0.001) and MFM scores (*p* = 0.030) improved significantly. There were neither SAEs nor deaths reported during the study period [56].

A multi-center prospective observational cohort study by Sansone et al. (2020) was conducted to evaluate the effectiveness of nusinersen on respiratory outcomes and ventilatory support in children with Type 1 SMA across tertiary neuromuscular centers in Italy. A total of 118 with Type 1 SMA were enrolled, stratified using Dubowitz’s decimal classification into subtypes 1.1 (n = 12), 1.5 (n = 65), and 1.9 (n = 38). Particularly among patients who received treatment < 2 years old, over 80% remained alive without requiring invasive mechanical ventilation or ≥16 h/day of NIV, which is significantly higher than the <10% survival among historical cohorts (*p* < 0.001). There were three patients who improved from >10 to ≤10 h of NIV/day, all with milder phenotypes (1.5 and 1.9). No patient with the severe 1.1 phenotype showed respiratory improvement. At 10 months of follow-up, 42.9% of patients who were initially on spontaneous breathing continued to breathe independently. Rates of respiratory infection (~33–38%; *p* > 0.05) and use of mechanical insufflation–exsufflation remained stable across follow-up visits. Qualitative interviews with caregivers revealed subjective improvements in respiratory function in 37% of the patients [57].

A single-center retrospective observational cohort study by Iwayama et al. (2022) was conducted to evaluate the effectiveness of nusinersen in adolescent and adult patients with Type 1 SMA and Type 2 SMA in Japan. A total of seven patients were enrolled, with a mean age of 23 years (range 12–40), either Type 1 SMA (n = 1) or Type 2 SMA (n = 6), although three Type 2 SMA patients were reclassified as Type 1c SMA due to early-onset symptoms and failure to achieve independent sitting. Patients generally received 11–14 doses of nusinersen over a median follow-up period of 3.55 years (range 1.78–4.53). Motor function scores increased over time, in terms of CHOP-INTEND scores from a median of 5.0 to 21.0 (median increase of 5.0; *p* = 0.10) and RULM scores from a median of 1.0 to 3.0 (median increase of 1.0; *p* = 0.089), though not statistically significant. HFMSE did not change significantly (*p* > 0.05). Only CHOP-INTEND scores showed clinically meaningful change (increase in score by ≥4), but still did not reach statistical significance. Two patients showed a decline in RULM scores, but this was attributed to delays in treatment due to COVID-19 restrictions [58].

A multi-center retrospective observational cohort study by Gonski et al. (2023) studied the respiratory and sleep outcomes in children with SMA treated with nusinersen over a four-year period, specifically two years before and two years after treatment initiation. Data were extracted from medical records, including spirometry, polysomnography (PSG), use of NIV, and motor function assessments using CHOP-INTEND, HFMSE, and WHO motor milestones. A total of 48 patients, either SMA Types 1 (n = 10), 2 (n = 23), or 3 (n = 15), aged 0.54 to 8.9 years at first dose. There were significant improvements in the baseline blood oxygen saturation during sleep (mean increase from 87.9% to 92.3%; mean difference of 4.4% [95% CI 1.24–7.63]; *p* = 0.01) and significant reductions in obstructive apnea–hypopnea index (decreased from 14.66 to 5.35 events/hr; *p* = 0.04) and total apnea–hypopnea index (*p* = 0.03). Six children, five of whom with Type 2 SMA and one with Type 3, stopped nightly use of NIV after nusinersen initiation, whereas the three others still required it post-treatment. There were significant increases in absolute FVC (1.37 L to 1.55 L; *p* = 0.01); however, there were no statistically significant changes in FVC% predicted and FVC z-score (*p* > 0.05). There were significant motor improvements among those with Type 1 SMA in terms of CHOP-INTEND scores (mean increase of 17; *p* = 0.02) and WHO scores (*p* = 0.02). There were no significant differences in the prevalence of respiratory hospitalization and length of stay [59].

A multi-center retrospective observational cohort study by Audic et al. (2024) evaluated the long-term clinical outcomes of nusinersen in children with Type 1 and Type 2 SMA, based on SMN2 copy number. Patients who initiated intrathecal nusinersen before the age of 3 years and completed at least 3 years of treatment without interruptions were enrolled. A total of 57 patients, either SMA Type 1 (n = 32) or 2 (n = 25). After 3 years of uninterrupted treatment, 93% of patients achieved new motor skills, more likely among those with three SMN2 copies; these include sitting (97%), crawling or rolling over (78%), standing with (62%) or without help (32%), and walking with (30%) or without assistance (14%). Contrastingly, none of the patients with two SMN2 copies achieved walking, and noticeably, only 20% could stand, but with help. Further, nutritional (75% vs. 11%) and ventilatory (60% vs. 5%) support requirements were higher among those with two SMN2 copies, compared to those with three copies. Likewise, orthopedic complications, particularly scoliosis, were more likely among those with two copies (100%) than those with three copies (64%) [60].

A single-center retrospective observational cohort study by Ergenekon et al. (2022) was conducted to examine the effectiveness of nusinersen in patients with Type 1 SMA in Istanbul, Turkey. Patients who completed four initial intrathecal nusinersen treatments and with follow-up visits of at least 180 days were enrolled. A total of 52 Type 1 SMA patients were enrolled, stratified into two groups based on the age when they received their first dose of treatment: ≤6 months (n = 19) and >6 months (n = 33). At the 180-day follow-up visit, 88.4% of patients remained alive. The overall survival did not significantly differ between groups (*p* = 0.65). Nonetheless, those who received treatment ≤ 6 months of age showed significantly better respiratory outcomes compared to those who received treatment at >6 months of age, in terms of higher proportion breathing spontaneously (43.7% vs. 13.3%; *p* = 0.03) and a lower rate of invasive mechanical ventilation (37.5% vs. 73.3%; *p* = 0.01). Both groups had significant improvements in CHOP-INTEND scores (median increase from 9.5 to 25.0; *p* < 0.001), though those who received treatment ≤ 6 months of life had higher gains (median score at day 180, 31.0 vs. 14.0; *p* = 0.01). Lastly, mortality was comparable (12.5% vs. 11.7%; *p* = 0.65) [61].

A single-center prospective observational cohort study by Arslan et al. (2022) studied the effectiveness and safety of nusinersen in adult patients with Type 2 and Type 3 SMA in Turkey. Patients aged >18 years, with genetically confirmed 5q-SMA with ≥2 SMN2 copies, and who completed four loading doses of nusinersen were enrolled. Motor function was assessed using HFMSE, Amyotrophic Lateral Sclerosis Functional Rating Scale—Revised (ALSFRS-R), Medical Research Council sum score (MRC-SS), and 6MWT, at baseline and 9-month and 15-month follow-up visits. A total of 32 patients were enrolled, 23 were males, median age 33.5 years (range 20–60), either SMA Type 2 (n = 6) or 3 (n = 26). There were significant improvements in HFMSE scores at 9 months (median increase of 3.0 points; *p* < 0.001) and 15 months (median increase of 4.0 points; *p* < 0.001). At 15-month follow-up, sitters with Type 2 SMA showed a gain of 2 points (*p* = 0.041). Among ambulatory patients, 6MWT significantly improved by a median of 20.0 m (*p* = 0.008). Overall, 78% of the patients had clinically significant responses (≥3-point HFMSE or ≥30 m 6MWT gains). AEs were mostly mild and self-limiting, and none led to discontinuation of treatment, including post-lumbar puncture headache (43.8%), injection site pain (53.1%), and proteinuria (32.2%) [62].

#### 2.3.2. Risdiplam

A national retrospective study by Belančić et al. (2024) included patients with SMA who previously received at least six doses of nusinersen, reimbursed by the CHIF, before switching to risdiplam with no relevant pause, and whose prespecified variables were available. Using the CHIF database and documentation of reimbursement, data collected included age during the switch, sex, SMA type and stage, SMN1/SMN2/Neuronal Apoptosis Inhibitory Protein (NAIP) copy numbers, number of nusinersen doses received, reasons for switching, risdiplam dosage and reimbursement requests, and motor function scores, particularly CHOP-INTEND for non-sitters/infants, HFFMSE for sitters and ambulant children, or RHS for ambulant adults. A total of 17 patients were included, 58.9% female, with a median age of 12.8 years (range 3.0–44.5), among whom n = 6 were Type 1 SMA, n = 4 were Type 3p SMA, and n = 7 were Type 3a SMA. At baseline, three patients required MV and three required BiPAP, all of whom had Type 1 SMA. A total of four Type 1 SMA patients required nutritional support, two using PEG and two using a nasogastric tube. Treatment switching was mostly indicated by difficulties in lumbar puncture (41.2%) and post-dural puncture headaches (11.8%). One year post-shift, risdiplam was non-inferior to nusinersen in all three subgroups in terms of motor function. Particularly, among patients with Type 1 SMA, CHOP-INTEND changed from 33.7 ± 23.7 to 34.7 ± 23.3 (*p* = 0.067). No patients experienced death or deterioration in terms of feeding and respiration [63].

A multi-center, retrospective observational cohort study by Cornell et al. (2024) was conducted to evaluate the short-term safety profile among pediatric patients with Types 1 and 2 SMA in Great Britain, accessing treatment through the Early Access to Medicines Scheme. Children aged at least 2 months with genetically confirmed 5q, Type 1 or 2 SMA, who were not indicated for any other authorized treatments during that time, were enrolled. Effectiveness assessments were not conducted. A total of 92 patients were enrolled, mean age of 10.9 years (range 0–17 years), either Type 1 (n = 20) or Type 2 (n = 72); among them, 56 were treatment-naive and 33 had previously received a DMD, mostly nusinersen. The median treatment duration was 11 months. There were 60 AEs reported in 34 patients; 38 (60%) were concluded to be unrelated to risdiplam, 10 (16%) were possibly related, and 12 (20%) had unspecified causality. The most common AEs were respiratory tract infections (35%), gastrointestinal disturbance (20%), and skin or hair issues (10%). Among the risdiplam-related events, 70% were gastrointestinal. There were four life-threatening events and two deaths in the study, though all were concluded to be unrelated to risdiplam. One patient permanently discontinued treatment due to irregularities in menstruation. There were no new safety signals, and the study confirmed a safety profile that is consistent with clinical trial findings [64].

A single-center retrospective observational cohort study by Sitas et al. (2024) studied the effectiveness and safety of risdiplam in adult patients with Type 2 and Type 3 SMA in Croatia. Patients who had one to four copies of the SMN2 gene and received risdiplam 5 mg daily for at least one year were enrolled. A total of 31 treatment-naive adult patients with either Type 2 SMA (n = 15) or Type 3 SMA (n = 16) were enrolled, with a median age of 30 years (range: 18–65) and a median duration of disease of 29 years. Among 31 patients, 22.6% of patients achieved clinically meaningful improvement (≥3-point gain) on at least one motor scale, with 61% experiencing stabilization of motor function. More than half (59%) of Type 2 SMA patients reported improvement in bulbar function, while all patients reported improved quality of life through the Individualized Neuromuscular Quality of Life scale. AEs were mild and self-limiting, including aphthous ulcers, headache, and transient laboratory abnormalities. No new safety signals were identified. Around a third of the cohort noted a weight gain of >5% along with reports of increased appetite or improved digestion [65].

A multi-center retrospective observational cohort study by Hahn et al. (2022) was conducted to evaluate the safety of risdiplam in patients with SMA, due to ineligibility, intolerance, or non-response to nusinersen or onasemnogene abeparvovec in a compassionate use program in Germany. Patients who were at least 2 months old, genetically confirmed with 5q-associated Type 1 or Type 2 SMA, and who had completed appropriate washout periods from prior treatments, were enrolled. A total of 111 patients were enrolled, either SMA Type 1 (n = 31) or 2 (n = 80), who received at least one dose of risdiplam. Most commonly reported AEs were gastrointestinal disorders. For Type 1 SMA, 30 events occurred among 13 patients, with 2 SAEs in one patient. For Type 2 SMA, 100 events occurred in 31 patients, including 8 SAEs in two patients. There were neither new safety signals nor fatal events. The most common SAEs included pneumonia and worsening of pre-existing gastrointestinal issues. Effectiveness data were not available due to regulations surrounding the compassionate use program [66].

A multi-center cohort study by Ashrafi et al. was conducted to compare risdiplam vs. nusinersen in children aged 2 to 10 years with genetically confirmed Type 2 and 3 SMA. Patients were assigned to receive either nusinersen or risdiplam based on clinical criteria or parental choice, with exclusion of those who had received other SMA therapies in the previous four months or had significant comorbidities. All participants underwent standardized motor function assessments using the HFMSE and the RULM at baseline, three months, and six months. A clinically meaningful response was defined as an increase of at least 3 points in either HFMSE or RULM. Among the 125 children included, none had Type 1 SMA, and all had either Type 2 or 3 SMA. In total, 52 (41.6%) patients received nusinersen and 73 (58.4%) received risdiplam. In both treatment groups, there was a statistically significant improvement in mean HFMSE and RULM scores at both three and six months compared to baseline (*p* < 0.001 for all comparisons). Specifically, after six months, 72% of patients in the nusinersen group and 80.4% of those in the risdiplam group achieved at least a 3-point increase in HFMSE or RULM, reflecting a robust motor response in both cohorts. However, there was no statistically significant difference in the magnitude of improvement between the two groups (*p* = 0.33 for the 3-point responder rate). No deaths or SAEs were reported in either group during the study period [67].

#### 2.3.3. Onasemnogene Abeparvovec

A single-center case series by Ali et al. (2021) was conducted to evaluate the safety and effectiveness of onasemnogene abeparvovec gene therapy in children with SMA in Qatar. Patients < 2 years old with genetically confirmed 5q SMA and bi-allelic SMN1 mutation and who were not on continuous invasive ventilation or who were not severely paralyzed were enrolled. A total of nine patients, either Type 1 (n = 7) or Type 2 (n = 2), with ages ranging from 4 to 23 months, were treated between November 2019 and July 2020 at Hamad Medical Corporation. After a single intravenous infusion of onasemnogene abeparvovec, patients were monitored every week for laboratory abnormalities and changes in motor outcomes. Patients with CHOP-INTEND scores at baseline showed significant motor improvement post-treatment (mean +11.8; range 7–18; paired *t*-test *p* = 0.0015). Transient abnormalities were common, yet patients were asymptomatic, including elevated AST or ALT (100%), thrombocytopenia (44.4%), troponin I (44.4%), prothrombin time (22.2%), and bilirubin (11.1%), and all were managed conservatively with corticosteroids. There was one reported case of transient vomiting after infusion [68].

A retrospective observational cohort study by Artemyeva et al. (2022) evaluated the safety and effectiveness of onasemnogene abeparvovec in children with genetically confirmed 5q SMA treated at the Research Clinical Pediatric Institute of Pirogov Russian National Research Medical University in Russia. A total of 41 patients, either Type 1 (n = 31) or Type 2 SMA (n = 10), with ages ranging from 5 to 47 months and all weighing ≤21 kg, received a single intravenous infusion of onasemnogene abeparvovec. Motor function was assessed using CHOP-INTEND and HINE-2 scales at baseline and at 6 and 12 months post-treatment. Among 17 patients with a 6-month follow-up, CHOP-INTEND scores increased by a mean score of +7.1 (*p* < 0.05) and HINE-2 by +3.3 (*p* < 0.05). Among 10 patients with a 12-month follow-up, the increases in CHOP-INTEND and HINE-2 were +9.4 (*p* < 0.05) and +4.4 (*p* < 0.01), respectively. AEs were commonly reported but were mostly mild and manageable, including elevated AST or ALT (78%), thrombocytopenia (22%), and short-term symptoms such as pyrexia, vomiting, and appetite loss in nearly all patients. One patient was reported to have experienced drug-induced liver injury, which required intensive corticosteroids and supportive management [69].

A longitudinal observational cohort study by Bitetti et al. (2024) was conducted to evaluate changes in motor and neurocognitive function over 12 months with onasemnogene abeparvovec in symptomatic children with Type 1 SMA and two SMN2 copies at Azienda Ospedaliera Universitaria della Regione Campania, in Naples, Italy. Patients weighing <13.5 kg, and with no contraindications to gene therapy per EMA criteria, were enrolled. The CHOP-INTEND scale was used to measure motor function at baseline and then at 1, 3, 6, and 12 months post-treatment; the Griffiths III scale was used to measure neurocognitive development at baseline, 6, and 12 months. A total of 12 patients, aged 28.0 (range 1.7–52.6) months, 9 of whom had previously received nusinersen, were enrolled. CHOP-INTEND scores significantly increased at all time points from baseline, with a mean increase of +32.4 points among those who were not previously treated with nusinersen and +11.2 points among those who were; 91.7% achieved the ability to sit or roll unassisted by 12 months. Griffiths III subscale scores in learning, coordination, communication, and socioemotional domains improved significantly, although most of the patients remained within the delayed or borderline delayed developmental range. However, language development was noted to be advanced in most patients, with half of the patients being able to speak in sentences at 12 months. There were no unexpected or severe AEs, with few exceptions, such as elevation in liver enzymes, which was managed with corticosteroids [70].

A single-center, longitudinal observational cohort study by de Holanda Mendonça et al. (2021) evaluated the safety and effectiveness of nusinersen in patients with Type 1 SMA at the Neuromuscular Clinic of Hospital das Clínicas, Faculty of Medicine, University of São Paulo in São Paulo, Brazil. A total of 21 patients were enrolled, all with Type 1 SMA, 52.4% male, with either two (n = 18) or three (n = 3) copies of the SMN2 gene. All patients gained increases in their CHOP-INTEND scores by a mean of 4.9 at 6 months, 5.9 at 12 months, 6.6 at 18 months, and 17.0 points at 24 months. Improvements were significantly associated with shorter duration of the disease (*p* = 0.006) and the absence of invasive mechanical ventilation at baseline (*p* = 0.018). Further, 28.6% of patients gained at least 3.0 points in the HINE-2 or achieved a new motor milestone, for instance, head control or independent sitting. Multivariable analysis found female sex (*p* = 0.021), shorter disease duration (*p* = 0.006), and use of non-invasive rather than invasive ventilation at baseline (*p* = 0.012) as significant predictors of treatment response in terms of improvement in HINE-2 scores. Among patients with NIV at baseline (n = 7), five experienced a reduction in daily ventilation time. Contrastingly, patients on invasive MV (n = 14) experienced minimal to no change in respiratory support requirements. AEs were minimal, including post-lumbar puncture headaches reported in 2.4% and one case of temporary intubation following sedation for intrathecal administration [71].

A prospective observational cohort study by D’Silva et al. (2022) evaluated the safety and effectiveness of onasemnogene abeparvovec in infants with SMA treated at the Sydney Children’s Hospital Network in Australia between August 2019 and November 2021. A total of 21 patients were enrolled, either non-sitters (n = 14, 66.7%), sitters (n = 3, 14.3%), or walkers (n = 2, 9.5%), ages ranging from 0.65 to 24 months, and weights ranging from 2.5 to 12.5 kg. Overall, 16 patients (76%) achieved at least one new WHO motor milestone, and 9/13 (69%) assessed with CHOP-INTEND or HFMSE experienced functional improvement over 6 months, with an average increase in scores by 7 and 10, respectively. Bulbar and respiratory functions remained the same or improved in 95.2% of patients. AEs were common but were mostly transient and manageable. Particularly, all patients experienced vomiting, 57% had transaminitis, and 33% had thrombocytopenia, more frequently and severe among children weighing ≥8 kg (*p* < 0.05). Two cases of thrombotic microangiopathy were noted, both among patients who were previously treated with nusinersen; they presented with vomiting, thrombocytopenia, hemolytic anemia, transaminitis, and acute kidney injury. There were neither serious nor long-term hepatic complications [72].

A single-center observational cohort study by Favia et al. (2024) evaluated the effectiveness and safety of onasemnogene abeparvovec in patients with Type 1 SMA at the Fondazione Policlinico Universitario Agostino Gemelli, Istituto di Ricovero e Cura a Carattere Scientifico, in Rome, Italy, treated between 2021 and 2023. A total of eight patients were included, with six patients previously treated with nusinersen. Among the eight patients, five showed clinical improvement defined as a ≥4-point increase in CHOP-INTEND scores, and one patient maintained a score of 64 throughout the follow-up period. There were improvements in other motor functions, including head control in three patients, independent sitting in five patients, and ambulation in one patient. Further, there was one patient who discontinued NIV at the end of the study. No AEs were observed, while one death occurred, but was assessed to be unrelated to the study treatment [73].

A study by Lavie et al. (2024) utilized a retrospective cohort design to characterize the natural history of Type 1 and 2 SMA. Patients were identified based on clinical and genetic diagnosis, with data extracted from medical records to assess motor milestones, respiratory support needs, and survival outcomes. Longitudinal follow-up allowed for evaluation of disease progression over time. Survival analyses were performed using Kaplan–Meier methods to estimate survival probabilities at various ages. Motor function was assessed using standardized scales, and correlations with genetic factors such as SMN2 copy number were examined. The study included 25 genetically confirmed SMA patients (23 with Type 1 and 2 with Type 2), with a median age at onasemnogene abeparvovec administration of 6.1 months. Sixteen patients were treatment-naïve, while nine had previously received disease-modifying therapies such as nusinersen or risdiplam. At baseline, prior to onasemnogene abeparvovec administration, 24% of patients (6/25) were receiving NIV, with three on permanent and three on partial support, averaging 14.3 h of ventilation daily. No patients required chronic invasive ventilation at this stage. Additionally, 48% utilized daily mechanical insufflation–exsufflation (MIE), and the respiratory-related hospitalization rate was 0.76 per life year. Most patients (80%) were able to maintain full oral nutrition at baseline. After one year of follow-up, two patients had died from respiratory failure, leaving 23 survivors for analysis. Within this cohort, four additional patients required initiation of NIV, raising the proportion of ventilated patients to 43% (10/23), with three necessitating permanent support. The mean daily duration of ventilation decreased to 11.1 h. Use of MIE increased slightly, with 16 of 23 survivors using it (12 daily, four during exacerbations). Importantly, the frequency of respiratory-related hospitalizations declined by 26%, from 0.76 to 0.57 per life year, indicating a clinically meaningful reduction in acute respiratory morbidity. No patients required chronic invasive ventilation at one year. Nutritional status showed some decline, with the number of patients maintaining full oral nutrition decreasing from 20 at baseline to 15 at follow-up, and four additional patients requiring gastrostomy placement due to feeding difficulties or aspiration. Subgroup analysis of the treatment-naïve cohort (n = 16, of whom 14 had Type 1 SMA and two had Type 2) revealed similar trends. Among the 12 surviving treatment-naïve Type 1 SMA patients, the proportion requiring NIV increased from 19% (3/16) at baseline to 43% (6/14) at one year, but the mean daily ventilation time decreased from 16 to 11.6 h. The use of MIE also increased, and the respiratory hospitalization rate in this subgroup decreased dramatically, from 2.1 to 0.75 per life year—a 64% reduction. Nutritional support needs increased, with four additional patients requiring gastrostomy during follow-up. In the two patients with Type 2 SMA, both were alive at one year post-onasemnogene abeparvovec and were included in the overall analysis, but specific respiratory outcomes for this subgroup were not separately detailed due to the small sample size. No acute respiratory complications or medication-related AEs were reported in association with onasemnogene abeparvovec administration. Mortality during the follow-up period was attributed to respiratory failure in two patients with Type 1 SMA, underscoring the ongoing vulnerability of this population despite advanced therapy [74].

A prospective, multi-center, observational cohort investigation by McMillan et al. (2024) was designed to assess the long-term outcomes of patients with Type 1 and 2 SMA who received DMDs. Eligible participants were genetically confirmed SMA patients, stratified by SMA type, and were enrolled from multiple specialized neuromuscular centers. The cohort included both treatment-naïve patients and those previously exposed to other SMA-directed therapies. Interventions consisted of administration of one or more approved disease-modifying agents, including nusinersen, onasemnogene abeparvovec, or risdiplam, following standard clinical protocols. Clinical assessments were conducted at baseline and at regular intervals post-treatment initiation, with a minimum follow-up period of 12 months. The primary endpoints included changes in motor function, evaluated using validated scales such as the CHOP-INTEND for Type 1 SMA and the HFMSE for Type 2 SMA. Secondary endpoints encompassed respiratory support requirements, nutritional status, and the incidence of AEs. The study cohort comprised a total of 100 patients, of whom 60 were diagnosed with Type 1 SMA and 40 with Type 2 SMA. The median age at treatment initiation was 5.2 months for Type 1 and 18.4 months for Type 2 patients. At baseline, the majority of Type 1 patients exhibited profound motor impairment, with CHOP-INTEND scores reflective of severe neuromuscular weakness. Type 2 patients demonstrated moderate motor deficits, with baseline HFMSE scores indicating partial preservation of gross motor function. Following initiation of disease-modifying therapy, patients with Type 1 SMA exhibited significant improvements in motor function. The mean CHOP-INTEND score increased by 14.2 points at 12 months post-treatment compared to baseline (*p* < 0.001). Notably, 45% of Type 1 patients achieved the milestone of independent sitting, a developmental achievement rarely observed in the natural history of untreated Type 1 SMA. The need for permanent ventilatory support was reduced, with only 30% of Type 1 patients requiring non-invasive ventilation at 12 months, compared to 52% at baseline. No new cases of chronic invasive ventilation were reported during the follow-up period. Nutritional outcomes also improved, with a decrease in the proportion of patients requiring gastrostomy feeding from 40% at baseline to 28% at 12 months. In the Type 2 SMA cohort, motor function gains were similarly robust. The mean HFMSE score increased by 7.6 points at 12 months (*p* < 0.001), and 60% of patients achieved new motor milestones, including the ability to stand with support and, in some cases, take independent steps. Respiratory outcomes were favorable, with a reduction in the proportion of patients requiring nocturnal non-invasive ventilation from 25% at baseline to 10% at follow-up. Nutritional status remained stable, with only a slight increase in the need for supplemental feeding. AEs across both cohorts were consistent with the known safety profiles of the administered therapies. The most common AEs included transient transaminase elevations and mild, self-limited respiratory infections. No treatment-related deaths or cases of severe organ toxicity were observed [75].

A multinational retrospective cohort study by Goedeker et al. (2024) included 66 infants with genetically confirmed 5q SMA treated at or before 6 weeks of age across 12 academic centers in the U.S. and Australia from 2018 to 2023. Inclusion criteria required infants to have two to four SMN2 copies, be identified pre-symptomatically (via newborn screening, prenatal testing, or family history), and have a baseline motor assessment with CHOP-INTEND prior to treatment. Infants treated with nusinersen or onasemnogene abeparvovec were included, while those in interventional trials or with other conditions affecting motor development were excluded. Results demonstrated that all infants achieved independent sitting, and most attained independent walking, though significant differences were observed based on SMN2 copy number. Among the cohort, 35 infants had two SMN2 copies and 31 had three or more. Those with two SMN2 copies presented with lower baseline CHOP-INTEND scores and were more likely to have symptomatic findings before treatment initiation. While 100% of infants with three or more SMN2 copies walked independently, only 68% of those with two copies achieved this milestone, and just 26% walked within the typical developmental window. Eleven children with two SMN2 copies remained non-ambulatory at last follow-up (ages 22–48 months). Early motor milestone delays, particularly delayed sitting (≥9 months), strongly predicted later walking delays or failure to walk. Symptomatic status at treatment initiation was also associated with poorer motor outcomes. Treatment predominantly involved onasemnogene abeparvovec (71%), with the remainder receiving nusinersen; some infants received sequential or combination therapies due to emerging SMA symptoms after initial treatment. No patients required permanent ventilation or exclusive enteral nutrition, though a minority with two SMN2 copies required nocturnal non-invasive ventilation or supplemental enteral nutrition. The findings highlight that early treatment improves outcomes but that significant motor disability persists in infants with two SMN2 copies, underscoring the need for further research into earlier, combination, or prenatal therapies and non-SMN-targeted approaches [76].

A multi-center prospective observational cohort study by Tokatly Latzer et al. (2023) evaluated the safety and effectiveness of onasemnogene abeparvovec in children with SMA treated across four tertiary hospitals in Israel. Patients who received a single intravenous onasemnogene abeparvovec infusion between November 2019 and April 2021 were enrolled. Patients were followed for a median of 18 months, with assessments including motor function tests, CHOP-INTEND and HFMSE, laboratory monitoring, and evaluations of respiratory, feeding, and speech outcomes. A total of 25 patients were enrolled, either SMA Type 1 (n = 23) or 2 (n = 2), aged between 11 days and 23 months at the time of treatment. The median CHOP-INTEND scores increased by 13.0 points (IQR 8.0–20.0), with greater gains among those treated before 8 months of age (median increase of 18.0 vs. 8.0 points; *p* = 0.002). None experienced motor regression, and 80% attained the ability to sit, 36% to crawl, and 32% to walk. There were transient AEs, including elevated liver enzymes (52%), particularly among older patients (*p* = 0.001, vs. younger patients), elevated troponin I (88%), and thrombocytopenia (36%); none of these resulted in permanent sequelae. There was one patient who died from respiratory illness, but was deemed unrelated to study treatment [77].

A multi-center prospective observational cohort study by Weiß et al. (2022) studied the safety and effectiveness of onasemnogene abeparvovec among pediatric patients with SMA in 18 neuromuscular centers in Germany and Austria. Patients with genetically confirmed Type 1 or Type 2 SMA who received onasemnogene abeparvovec between September 2019 and April 2021 were enrolled, including those who were pre-treated with nusinersen. A total of 76 patients were enrolled, either Type 1 (n = 51), Type 2 (n = 19) SMA, or presymptomatic (n = 6), and 58 were previously treated with nusinersen. Among patients with available data on motor function (n = 60), 82% had clinically meaningful improvements, defined as an increase of ≥4 points on CHOP-INTEND or ≥3 points on HFMSE. The mean CHOP-INTEND scores increased by 13.8 points among children ≤ 8 months (*p* < 0.0001) and 7.7 points among those 8–24 months (*p* < 0.0001); CHOP-INTEND scores did not significantly increase among those > 24 months, with a change of 2.5 points (*p* = 1.00). Among those pre-treated with nusinersen (n = 45), patients gained a mean 8.8 increase in CHOP-INTEND post-switch (*p* < 0.0001), while treatment-naive patients (n = 11) gained 9.4 points (*p* = 0.003). Further, around half (54%) achieved at least one new motor milestone. Around 74% patients experienced AEs, including pyrexia (62%), vomiting or loss of appetite (54%), and thrombocytopenia (78%). SAEs were reported in 11% of the patients, including acute liver dysfunction (8%), more commonly among older or heavier patients (*p* < 0.0001) and those pre-treated with nusinersen (*p* < 0.0001) [78].

## 3. Discussion

### 3.1. Future Directions: Gene Modifiers and Predicting Therapy Outcome

Predicting therapeutic responses based on a multitude of personalized factors is one of the key goals of medicine in this century [79]. There is a multitude of gene therapies being developed right now, and in general, it is a growing topic that is evidenced by an increasing number of indexed publications [80]. The most obvious genetic modifier for clinical severity is the copy number of *SMN2*, which leads to the classical disease types in SMA [81]. However, individuals with the same amount of *SMN2* copies can have varying disease courses, especially in cases where *SMN1* is not deleted, meaning if a patient has a partial-function *SMN1* protein, that might mitigate severity regardless of *SMN2* count [82]. Aside from that, several other genetic modifiers have been identified that influence the phenotype in SMA. These include NAIP, Plastin-3 (PLS3), and Neurocalcin-δ (NCALD), which have been shown to modulate disease severity through various cellular pathways. Additionally, emerging evidence points to other modifier genes such as CHP1 and ZPR1, which may affect motor neuron survival and SMN protein function, respectively, offering promising targets for future therapeutic interventions. Further exploration of these and other modifier genes could help refine prognosis and personalize treatment strategies for SMA patients [83,84].

The absence of NAIP, which is common in Type 1, likely exacerbates the disease by removing a neuroprotective factor. An Egyptian study combining *SMN2* and NAIP genotypes found that NAIP deletion plus low *SMN2* was associated with the worst outcomes, while NAIP intact plus higher *SMN2* was associated with milder SMA [4]. Furthermore, female patients with naturally high PLS3 expression might have a milder starting point and possibly respond better to treatment due to a better state of motor neurons, while those with low PLS3 expression might have more synaptic vulnerability. One study found that, among females with 3 SMN2 copies on therapy, those who achieved walking had higher PLS3 expression than those who did not, indicating a modifying effect on clinical outcomes [85]. Finally, a modifier such as NCALD does not directly influence the action of treatment like nusinersen or risdiplam, but can affect the overall health of motor neurons. Even though there is no clinical evidence thus far, a research group showed that adding an NCALD-targeting ASO on top of nusinersen in mice yielded better motor neuron protection than nusinersen alone [86].

In summary, the genetic milieu in SMA can modulate treatment outcomes in ways beyond just SMN2 count. Most notably, rare *SMN2* splice variants can determine whether a patient responds to splicing modifiers at all [87], while modifiers like NAIP, PLS3, and others influence the baseline neuronal health and thus the degree of recovery possible. Recognizing these factors can help tailor expectations and, in the future, may guide adjunctive treatments to maximize each patient’s response.

### 3.2. Prenatal Therapy

Intrauterine prenatal therapy provides an interesting option for treating genetic disorders during the earliest development [88]. The field is in its infancy, and there are numerous questions that need an answer. There are many candidate diseases and therapies for prenatal therapy, including SMA [89]. The main problem is the way of administration and the timing, as research is required to see which vectors can be used for transfection [89]. Delivery methods pose substantial challenges, such as ensuring accurate targeting, minimizing maternal and fetal risks, and avoiding unwanted immune responses.

An interesting case report was published recently with onasemnogene abeparvovec administration in two prematurely born girls (30 weeks of gestation) at 3.5 weeks of life, with a normal neurological examination at 19 months. The authors posit that a gene modifier with early drug administration due to prematurity was responsible for the favorable outcomes [90]. Finally, a very recent case report highlighted the first use of prenatal therapy of SMA using risdiplam, now more than two years after birth, with no clinical features of the disease [91]. These two reports highlight the benefits of early treatment, but there are clear challenges ahead in implementing them, including screening and prenatal testing.

### 3.3. Combining Therapies

Current management of SMA increasingly involves multidimensional treatment strategies. Even with disease-modifying SMN-enhancing drugs, patients must also receive concurrent supportive intervention, such as physical therapy, nutritional support, and respiratory care, alongside disease-modifying therapy [92]. Some patients switch from nusinersen to oral risdiplam, or vice versa, due to convenience or suboptimal response, and studies have shown that initiating risdiplam in patients previously on nusinersen or gene therapy is safe and can maintain motor function [63]. Also, it is possible to combine onasemnogene abeparvovec by following it up with nusinersen or risdiplam, typically in patients with an incomplete response to initial therapy, with a phase 4 trial in place testing this [93]. It seems that the current trend in care is pointing towards combining different modalities of currently available treatment, even though it is clear that more research and clinical trials are needed.

### 3.4. Unmet Needs and Challenges

Unresolved issues in SMA treatment notably include challenges in managing older patients and those who exhibit limited or no response to current therapies.

Older individuals with SMA, particularly those beyond early childhood, often have fewer treatment options and may experience disease progression compounded by aging, which is not fully addressed by existing therapies.

Non-responders to current DMDs also represent a significant clinical challenge, necessitating alternative approaches. Several ongoing clinical trials are actively investigating combination therapies that integrate existing SMN-enhancing treatments with novel agents targeting SMN-independent pathways. These include therapies aimed at muscle function improvement, neuroprotection, and modulation of other cellular mechanisms involved in SMA pathology. Such approaches hold promise for broadening the therapeutic landscape to address these unmet needs and improve outcomes across different patient subgroups. Continued research and trial data will be critical to define efficacious strategies for these complex cases and to optimize individualized treatment plans [94,95,96,97].

Another aspect worth highlighting is that comparative data across the three disease-modifying therapies for SMA remain limited. As highlighted by a recently published systematic literature review focused specifically on studies examining treatment switching in SMA, consistent and comprehensive comparative analyses are scarce. The available evidence is largely based on a few real-world studies and clinical trials, with substantial variability in outcome measures and a lack of standardized data, making direct cross-drug comparisons challenging [98].

### 3.5. Glial Modulation as Potential Therapies

Experimental or investigational therapy combinations can include other treatment modalities other than gene therapy, knowing that modulating astrocyte and microglia has potential therapeutic implications in most neurodegenerative conditions, including SMA [99]. Research indicates that conditioned media from SMA astrocytes negatively affect motor neurons (MNs), resulting in reduced neurite length and diminished expression of MN-specific markers. This supports the hypothesis that dysfunctional astrocytes secrete factors contributing to MN degeneration. Additionally, the interaction between microglia and astrocytes may enhance neuroinflammatory processes in SMA, and abnormal synaptic pruning by microglia may further exacerbate motor neuron deafferentation, worsening the disease [100,101].

Recent advancements in astrocyte-specific gene delivery systems have utilized adeno-associated virus (AAV) vectors designed to express therapeutic genes selectively in astrocytes. Notably, astrocyte-targeted delivery of IL-2 has effectively modulated inflammatory responses without altering peripheral immunity [102]. Microglia-focused therapies aim to adjust microglial activity toward protective phenotypes. Strategies targeting microglial signaling pathways can enhance microglial homeostatic functions in neurodegeneration. Additionally, targeting specific microglial receptors has been linked to reduced chronic inflammation in conditions such as SMA [103,104]. One of the possible targets is synaptic removal, though acting on complement proteins like C1q, which is a critical element in the complement cascade [105]. Functionally, the type of intervention would be an immunotherapy approach, which highlights the possible combination of genetic therapies with monoclonal antibodies to further strengthen therapeutic response.

### 3.6. New Research Aims with Regards to Therapies

Looking ahead, research in SMA is converging on key themes to refine and extend therapeutic impact. As written above, the major focuses include earlier intervention, precision medicine, and therapy combinations that include gene, immune, and supportive therapies. Recently published pioneering case report regarding in vivo gene editing highlights the upcoming possibilities regarding treatment, which will surely be used in many genetic diseases [106]. Future research aims should be focused on overcoming key challenges such as the timing of therapy, delivery of medication, type of intervention, adequate timing of combining therapies, and characterizing new phenotypes that will emerge with long-term follow-up [107]. In summary, the future of therapy lies in optimization and integration, by refining therapies through precision medicine, combining agents to address both motor neuron dysfunction and its secondary effects, and innovating delivery technologies. By embracing these goals, it will be possible to further improve survival and quality of life for all individuals with SMA, moving closer to a paradigm where SMA is not only treatable but comprehensively managed for the long term [108].

## Figures and Tables

**Table 1:** The PICOS criteria followed for the conduct of this narrative review.

Population	Patients Diagnosed with Type 1 and Type 2 SMA Across All Age Groups
Intervention	Nusinersen; risdiplam; onasemnogene abeparvovec
Comparator	Nusinersen; risdiplam; onasemnogene abeparvovec
Outcome	Motor function; survival outcomes; quality of life
Study design	Clinical trials; observational studies; real-world studies

SMA, spinal muscular atrophy.

## Data Availability

No new data were created.

## Abbreviations

The following abbreviations are used in this manuscript:

6MWT	6-Minute Walking Test
AAV	Adeno-associated virus
AAV9	Adeno-associated virus serotype 9
AEs	Adverse events
ALSFRS-R	Amyotrophic Lateral Sclerosis Functional Rating Scale—Revised
ASO	Antisense oligonucleotide
BSID-III	Bayley Scales of Infant and Toddler Development, Third Edition
CHIF	Croatian Health Insurance Fund
CHOP-INTEND	Children’s Hospital of Philadelphia Infant Test of Neuromuscular Disorders
CI	Confidence interval
CMAP	Compound muscle action potential
CSF	Cerebrospinal fluid
DMDs	Disease-modifying drugs
EAP	Expanded Access Program
EMA	European Medicines Agency
FVC	Forced vital capacity
GFAP	Glial fibrillary acidic protein
GSI-I	Patient Global Impression of Improvement
HFMSE	Hammersmith Functional Motor Scale—Expanded
HINE-2	Hammersmith Infant Neurological Examination—Section 2
ISS-N1	Intronic splice silencing site
ITT	Intention to treat
LCI	Lung clearance index
MFM32	Motor Function Measure-32
MNs	Motor neurons
MRC-SS	Medical Research Council sum score
MUNE	Motor Unit Number Estimation
NAIP	Neuronal Apoptosis Inhibitory Protein
NCALD	Neurocalcin-δ
NFL	Neurofilament light
NIV	Non-invasive ventilation
PD	Pharmacodynamics
PEG	Percutaneous endoscopic gastrostomy
PK	Pharmacokinetics
PLS3	Plastin-3
PNCR	Pediatric Neuromuscular Clinical Research
PSG	Polysomnography
PT	Physical therapy
RCTs	Randomized controlled trials
RHS	Revised Hammersmith Scale
RULM	Revised Upper Limb Module
SAEs	Serious adverse events
SMA	Spinal muscular atrophy
SMN	Survival motor neuron
WHO	World Health Organization

## References

[1] Darras B.T., Markowitz J.A., Monani U.R., De Vivo D.C. (2015). Spinal Muscular Atrophies. Neuromuscular Disorders of Infancy, Childhood, and Adolescence.

[2] Verhaart I.E.C., Robertson A., Wilson I.J., Aartsma-Rus A., Cameron S., Jones C.C., Cook S.F., Lochmüller H. (2017). Prevalence, Incidence and Carrier Frequency of 5q–Linked Spinal Muscular Atrophy—A Literature Review. Orphanet J. Rare Dis..

[3] Shahram S., Ashrafi M.R., Niusha S., Heidari M., Mohammad-Hossein M., Zamani G., Saloomeh A., Sarang Y., Mahdi M., Pourandokht S. (2023). A Comprehensive Overview of SMN and NAIP Copy Numbers in Iranian SMA Patients. Sci. Rep..

[4] Hassan H.A., Zaki M.S., Issa M.Y., El-Bagoury N.M., Essawi M.L. (2020). Genetic Pattern of SMN1, SMN2, and NAIP Genes in Prognosis of SMA Patients. Egypt. J. Med. Hum. Genet..

[5] Chen T.-H. (2020). New and Developing Therapies in Spinal Muscular Atrophy: From Genotype to Phenotype to Treatment and Where Do We Stand?. Int. J. Mol. Sci..

[6] Talbot K., Tizzano E.F. (2017). The Clinical Landscape for SMA in a New Therapeutic Era. Gene Ther..

[7] Mercuri E., Finkel R.S., Muntoni F., Wirth B., Montes J., Main M., Mazzone E.S., Vitale M., Snyder B., Quijano-Roy S. (2018). Diagnosis and Management of Spinal Muscular Atrophy: Part 1: Recommendations for Diagnosis, Rehabilitation, Orthopedic and Nutritional Care. Neuromuscul. Disord..

[8] Finkel R.S., Mercuri E., Meyer O.H., Simonds A.K., Schroth M.K., Graham R.J., Kirschner J., Iannaccone S.T., Crawford T.O., Woods S. (2018). Diagnosis and Management of Spinal Muscular Atrophy: Part 2: Pulmonary and Acute Care; Medications, Supplements and Immunizations; Other Organ Systems; and Ethics. Neuromuscul. Disord..

[9] Tizzano E.F., Finkel R.S. (2017). Spinal Muscular Atrophy: A Changing Phenotype beyond the Clinical Trials. Neuromuscul. Disord..

[10] Farrar M.A., Carey K.A., Paguinto S.-G., Kasparian N.A., De Abreu Lourenço R. (2020). “The Whole Game Is Changing and You’ve Got Hope”: Australian Perspectives on Treatment Decision Making in Spinal Muscular Atrophy. Patient-Patient-Centered Outcomes Res..

[11] Sukhera J. (2022). Narrative Reviews: Flexible, Rigorous, and Practical. J. Grad. Med. Educ..

[12] Spinraza—European Medicines Agency European Medicines Agency. https://www.ema.europa.eu/en/medicines/human/EPAR/spinraza.

[13] Finkel R.S., Mercuri E., Darras B.T., Connolly A.M., Kuntz N.L., Kirschner J., Chiriboga C.A., Saito K., Servais L., Tizzano E. (2017). Nusinersen versus Sham Control in Infantile-Onset Spinal Muscular Atrophy. N. Engl. J. Med..

[14] Mercuri E., Darras B.T., Chiriboga C.A., Day J.W., Campbell C., Connolly A.M., Iannaccone S.T., Kirschner J., Kuntz N.L., Saito K. (2018). Nusinersen versus Sham Control in Later-Onset Spinal Muscular Atrophy. N. Engl. J. Med..

[15] Evrysdi—European Medicines Agency European Medicines Agency. https://www.ema.europa.eu/en/medicines/human/EPAR/evrysdi.

[16] Darras B.T., Masson R., Mazurkiewicz-Bełdzińska M., Rose K., Xiong H., Zanoteli E., Baranello G., Bruno C., Vlodavets D., Wang Y. (2021). Risdiplam-Treated Infants with Type 1 Spinal Muscular Atrophy versus Historical Controls. N. Engl. J. Med..

[17] Baranello G., Darras B.T., Day J.W., Deconinck N., Klein A., Masson R., Mercuri E., Rose K., El-Khairi M., Gerber M. (2021). Risdiplam in Type 1 Spinal Muscular Atrophy. N. Engl. J. Med..

[18] Mercuri E., Deconinck N., Mazzone E.S., Nascimento A., Oskoui M., Saito K., Vuillerot C., Baranello G., Boespflug-Tanguy O., Goemans N. (2022). Safety and efficacy of once-daily risdiplam in type 2 and non-ambulant type 3 spinal muscular atrophy (SUNFISH part 2): A phase 3, double-blind, randomised, placebo-controlled trial. Lancet Neurol..

[19] Zolgensma—European Medicines Agency European Medicines Agency. https://www.ema.europa.eu/en/medicines/human/EPAR/zolgensma.

[20] Day J.W., Finkel R.S., Chiriboga C.A., Connolly A.M., Crawford T.O., Darras B.T., Iannaccone S.T., Kuntz N.L., Peña L.D.M., Shieh P.B. (2021). Onasemnogene Abeparvovec Gene Therapy for Symptomatic Infantile-Onset Spinal Muscular Atrophy in Patients with Two Copies of SMN2 (STR1VE): An Open-Label, Single-Arm, Multicentre, Phase 3 Trial. Lancet Neurol..

[21] Mendell J.R., Al-Zaidy S., Shell R., Arnold W.D., Rodino-Klapac L.R., Prior T.W., Lowes L., Alfano L., Berry K., Church K. (2017). Single-Dose Gene-Replacement Therapy for Spinal Muscular Atrophy. N. Engl. J. Med..

[22] Mercuri E., Muntoni F., Baranello G., Masson R., Boespflug-Tanguy O., Bruno C., Corti S., Daron A., Deconinck N., Servais L. (2021). Onasemnogene Abeparvovec Gene Therapy for Symptomatic Infantile-Onset Spinal Muscular Atrophy Type 1 (STR1VE-EU): An Open-Label, Single-Arm, Multicentre, Phase 3 Trial. Lancet Neurol..

[23] Strauss K.A., Farrar M.A., Muntoni F., Saito K., Mendell J.R., Servais L., McMillan H.J., Finkel R.S., Swoboda K.J., Kwon J.M. (2022). Onasemnogene Abeparvovec for Presymptomatic Infants with Two Copies of SMN2 at Risk for Spinal Muscular Atrophy Type 1: The Phase III SPR1NT Trial. Nat. Med..

[24] Acsadi G., Crawford T.O., Müller-Felber W., Shieh P.B., Richardson R., Natarajan N., Castro D., Ramirez-Schrempp D., Gambino G., Sun P. (2021). Safety and Efficacy of Nusinersen in Spinal Muscular Atrophy: The EMBRACE Study. Muscle Nerve.

[25] Darras B.T., Chiriboga C.A., Iannaccone S.T., Swoboda K.J., Montes J., Mignon L., Xia S., Bennett C.F., Bishop K.M., Shefner J.M. (2019). Nusinersen in Later-Onset Spinal Muscular Atrophy. Neurology.

[26] Tachibana Y., Sato R., Makioka H., Hoshino M., Jin M. (2023). Safety and Effectiveness of Nusinersen, a Treatment for Spinal Muscular Atrophy, in 524 Patients: Results from an Interim Analysis of Post-Marketing Surveillance in Japan. Int. J. Neurosci..

[27] ClinicalTrials.Gov A Study to Investigate the Safety and Efficacy of RO7204239 in Combination with Risdiplam (RO7034067) in Participants with Spinal Muscular Atrophy (MANATEE). https://clinicaltrials.gov/study/NCT05115110.

[28] Chiriboga C.A., Bruno C., Duong T., Fischer D., Mercuri E., Kirschner J., Kostera-Pruszczyk A., Jaber B., Ksenija G., Heidemarie K. (2024). JEWELFISH: 24-Month Results from an Open-Label Study in Non-Treatment-Naïve Patients with SMA Receiving Treatment with Risdiplam. J. Neurol..

[29] Aragon-Gawinska K., Andreea M.S., Daron A., Gargaun E., Vuillerot C., Cances C., Ropars J., Mondher C., Cuppen I., Hughes I. (2018). Nusinersen in Patients Older than 7 Months with Spinal Muscular Atrophy Type 1. Neurology.

[30] Aragon-Gawinska K., Daron A., Ulinici A., Laura V.B., Seferian A., Gidaro T., Mariacristina S., Deconinck N., Servais L. (2019). Sitting in Patients with Spinal Muscular Atrophy Type 1 Treated with Nusinersen. Dev. Med. Child Neurol..

[31] Belančić A., Strbad T., Marta K.Š., Dinko V. (2023). Effectiveness of Nusinersen in Type 1, 2 and 3 Spinal Muscular Atrophy: Croatian Real-World Data. J. Clin. Med..

[32] Chan S.H.-S., Chae J.-H., Chien Y.-H., Ko T.-S., Lee J.H., Lee Y.J., Nam S.O., Jong Y.-J. (2021). Nusinersen in Spinal Muscular Atrophy Type 1 from Neonates to Young Adult: 1-Year Data from Three Asia-Pacific Regions. J. Neurol. Neurosurg. Psychiatry.

[33] Chen K.-A., Widger J., Teng A., Fitzgerald D.A., D’Silva A., Farrar M. (2021). Real-World Respiratory and Bulbar Comorbidities of SMA Type 1 Children Treated with Nusinersen: 2-Year Single Centre Australian Experience. Paediatr. Respir. Rev..

[34] Yao X., Peng J., Luo R., Wang X., Lu X., Wu L., Jin R., Zhong J., Liang J., Hong S. (2024). Nusinersen Effectiveness and Safety in Pediatric Patients with 5q-Spinal Muscular Atrophy: A Multi-Center Disease Registry in China. J. Neurol..

[35] Jiang Y., Wang Y., Xiong H., Li W., Luo R., Chen W., Yin F., Lü J., Liang J., Chen W.-J. (2024). A Post-Marketing Surveillance Study of Nusinersen for Spinal Muscular Atrophy in Routine Medical Practice in China: Interim Results. Adv. Ther..

[36] Wang N., Hu Y., Jiao K., Cheng N., Sun J., Tang J., Song J., Sun C., Wang T., Wang K. (2024). Long-Term Impact of Nusinersen on Motor and Electrophysiological Outcomes in Adolescent and Adult Spinal Muscular Atrophy: Insights from a Multicenter Retrospective Study. J. Neurol..

[37] Li D., Yang J., Wang X., Yang L., Luo R., Huang S. (2024). Analysis of the Efficacy and Adverse Effects of Nusinersen in the Treatment of Children with Spinal Muscular Atrophy in China. Brain Behav..

[38] Gaboli M., Lobato M.L., Fernández J.V., Ferri P.F., Pérez E.R., Ruiz H.A.A., González J.M.L.-P., Madruga-Garrido M. (2024). Effect of Nusinersen on Respiratory and Bulbar Function in Children with Spinal Muscular Atrophy: Real World Experience from a Single Center. Neuropediatrics.

[39] Axente M., Mirea A., Sporea C., Pădure L., Drăgoi C.M., Nicolae A.C., Ion D.A. (2022). Clinical and Electrophysiological Changes in Pediatric Spinal Muscular Atrophy after 2 Years of Nusinersen Treatment. Pharmaceutics.

[40] Chacko A., Sly P.D., Ware R.S., Begum N., Deegan S., Thomas N., Gauld L.M. (2022). Effect of Nusinersen on Respiratory Function in Paediatric Spinal Muscular Atrophy Types 1–3. Thorax.

[41] Cho J., Lee J., Kim J., Lee H., Kim M.-J., Lee Y.J., Yum M.-S., Byun J.-H., Lee C.G., Lee Y.-M. (2023). Nusinersen Demonstrates Effectiveness in Treating Spinal Muscular Atrophy: Findings from a Three-Year Nationwide Study in Korea. Front. Neurol..

[42] Osredkar D., Jílková M., Butenko T., Loboda T., Golli T., Fuchsová P., Rohlenová M., Haberlova J. (2021). Children and Young Adults with Spinal Muscular Atrophy Treated with Nusinersen. Eur. J. Paediatr. Neurol..

[43] Mirea A., Leanca M.C., Onose G., Sporea C., Padure L., Shelby E.-S., Dima V., Daia C. (2022). Physical Therapy and Nusinersen Impact on Spinal Muscular Atrophy Rehabilitative Outcome. Front. Biosci..

[44] Modrzejewska S., Kotulska K., Kopyta I., Grędowska E., Emich-Widera E., Tomaszek K., Paprocka J., Chmielewski D., Pilch J., Pietruszewski J. (2021). Nusinersen Treatment of Spinal Muscular Atrophy Type 1—Results of Expanded Access Programme in Poland. Neurol. I Neurochir. Pol..

[45] Olsson B., Alberg L., Cullen N.C., Michael E., Wahlgren L., Kroksmark A.-K., Rostasy K., Blennow K., Zetterberg H., Tulinius M. (2019). NFL Is a Marker of Treatment Response in Children with SMA Treated with Nusinersen. J. Neurol..

[46] Pane M., Coratti G., Sansone V.A., Messina S., Catteruccia M., Bruno C., Sframeli M., Albamonte E., Pedemonte M., Brolatti N. (2023). Type I Spinal Muscular Atrophy Patients Treated with Nusinersen: 4-Year Follow-up of Motor, Respiratory and Bulbar Function. Eur. J. Neurol..

[47] Pechmann A., Langer T., Schorling D., Stein S., Vogt S., Schara U., Kölbel H., Schwartz O., Hahn A., Giese K. (2018). Evaluation of Children with SMA Type 1 under Treatment with Nusinersen within the Expanded Access Program in Germany. J. Neuromuscul. Dis..

[48] Szabó L., Gergely A., Jakus R., Fogarasi A., Grosz Z., Molnár M.J., Andor I., Schulcz O., Goschler Á., Medveczky E. (2020). Efficacy of Nusinersen in Type 1, 2 and 3 Spinal Muscular Atrophy: Real World Data from Hungarian Patients. Eur. J. Paediatr. Neurol..

[49] Tscherter A., Rüsch C.T., Baumann D., Enzmann C., Hasselmann O., Jacquier D., Jung H.H., Kruijshaar M.E., Kuehni C.E., Neuwirth C. (2022). Evaluation of Real-Life Outcome Data of Patients with Spinal Muscular Atrophy Treated with Nusinersen in Switzerland. Neuromuscul. Disord..

[50] Weststrate H., Stimpson G., Thomas L., Scoto M., Johnson E., Stewart A., Muntoni F., Baranello G., Conway E., Manzur A. (2022). Evolution of Bulbar Function in Spinal Muscular Atrophy Type 1 Treated with Nusinersen. Dev. Med. Child Neurol..

[51] Günther R., Wurster C.D., Brakemeier S., Osmanovic A., Schreiber-Katz O., Petri S., Uzelac Z., Hiebeler M., Thiele S., Walter M.C. (2024). Long-Term Efficacy and Safety of Nusinersen in Adults with 5q Spinal Muscular Atrophy: A Prospective European Multinational Observational Study. Lancet Reg. Health-Eur..

[52] Hully M., Barnerias C., Chabalier D., Le Guen S., Germa V., Deladriere E., Vanhulle C., Cuisset J.-M., Chabrol B., Cances C. (2020). Palliative Care in SMA Type 1: A Prospective Multicenter French Study Based on Parents’ Reports. Front. Pediatr..

[53] Łusakowska A., Wójcik A., Frączek A., Aragon-Gawińska K., Potulska-Chromik A., Baranowski P., Nowak R., Rosiak G., Milczarek K., Konecki D. (2023). Long-Term Nusinersen Treatment across a Wide Spectrum of Spinal Muscular Atrophy Severity: A Real-World Experience. Orphanet J. Rare Dis..

[54] Yang H., Tao Q., Li D., Yang J., Cai Q., Gan J., Huang S., Luo R. (2023). Assessment of Motor Function and Nutritional Status in Children with Spinal Muscular Atrophy Treated with Nusinersen after Loading Period in Western China: A Retrospective Study. BMC Neurol..

[55] Kotulska K., Chmielewski D., Mazurkiewicz-Bełdzińska M., Tomaszek K., Pierzchlewicz K., Rabczenko D., Przysło Ł., Biedroń A., Czyżyk E., Steinborn B. (2022). Safety, Tolerability, and Efficacy of a Widely Available Nusinersen Program for Polish Children with Spinal Muscular Atrophy. Eur. J. Paediatr. Neurol..

[56] Gómez-García M.B., Alessandro A., Khirani S., Sandrine P., Barnérias C., Dabaj I., Bénézit A., Durigneux J., Carlier R., Desguerre I. (2020). Assessment of Respiratory Muscles and Motor Function in Children with SMA Treated by Nusinersen. Pediatr. Pulmonol..

[57] Sansone V.A., Pirola A., Albamonte E., Pane M., Lizio A., D’Amico A., Catteruccia M., Cutrera R., Bruno C., Pedemonte M. (2020). Respiratory Needs in Patients with Type 1 Spinal Muscular Atrophy Treated with Nusinersen. J. Pediatr..

[58] Iwayama H., Kawahara K., Takagi M., Numoto S., Azuma Y., Kurahashi H., Yasue Y., Kawajiri H., Yanase A., Ito T. (2023). Long-Term Efficacy of Nusinersen and Its Evaluation in Adolescent and Adult Patients with Spinal Muscular Atrophy Types 1 and 2. Brain Dev..

[59] Gonski K., Chuang S., Teng A., Thambipillay G., Farrar M., Menezes M., Fitzgerald D. (2023). Respiratory and Sleep Outcomes in Children with SMA Treated with Nusinersen-Real World Experience. Neuromuscul. Disord..

[60] Audic F., Dubois S.M., Durigneux J., Barnerias C., Isapof A., Nougues M.-C., Davion J.-B., Richelme C., Vuillerot C., Legoff L. (2023). Effect of Nusinersen after 3 Years of Treatment in 57 Young Children with SMA in Terms of SMN2 Copy Number or Type. Arch. Pédiatrie.

[61] Ergenekon A.P., Yegit C.Y., Cenk M., Gokdemir Y., Eralp E.E., Ozturk G., Unver O., Coskun O.K., Saygi E.K., Turkdogan D. (2022). Respiratory Outcome of Spinal Muscular Atrophy Type 1 Patients Treated with Nusinersen. Pediatr. Int..

[62] Arslan D., Inan B., Kilinc M., Bekircan-Kurt C.E., Erdem-Ozdamar S., Tan E. (2023). Nusinersen for Adults with Spinal Muscular Atrophy. Neurol. Sci..

[63] Belančić A., Strbad T., Štiglić M.K., Vitezić D. (2024). Switching from Nusinersen to Risdiplam: A Croatian Real-World Experience on Effectiveness and Safety. J. Pers. Med..

[64] Cornell N., Childs A.-M., Wraige E., Munot P., Ambegaonkar G., Chow G., Hughes I., Illingworth M., Majumdar A., Marini-Bettolo C. (2024). Risdiplam in Spinal Muscular Atrophy: Safety Profile and Use through the Early Access to Medicine Scheme for the Paediatric Cohort in Great Britain. J. Neuromuscul. Dis..

[65] Sitaš B., Hančević M., Bilić K., Bilić H., Bilić E. (2024). Risdiplam Real World Data—Looking beyond s and Motor Function Measures. J. Neuromuscul. Dis..

[66] Hahn A., Günther R., Ludolph A., Schwartz O., Trollmann R., Weydt P., Weiler M., Neuland K., Schwaderer M.S., Hagenacker T. (2022). Short-Term Safety Results from Compassionate Use of Risdiplam in Patients with Spinal Muscular Atrophy in Germany. Orphanet J. Rare Dis..

[67] Ashrafi M.R., Babaee M., Nazari S.S.H., Barzegar M., Ghazavi M., Toosi M.B., Nafissi S., Inaloo S., Ghaletaki G.Z., Fatehi F. (2024). Comparative Efficacy of Risdiplam and Nusinersen in Type 2 and 3 Spinal Muscular Atrophy Patients: A Cohort Study Using Real-World Data. J. Neuromuscul. Dis..

[68] Ali H.G., Ibrahim K., Elsaid M.F., Mohamed R.B., Abeidah M.I.A., Al Rawwas A.O., Elshafey K., Almulla H., El-Akouri K., Almulla M. (2021). Gene Therapy for Spinal Muscular Atrophy: The Qatari Experience. Gene Ther..

[69] Artemyeva S.B., Papina Y.O., Shidlovskaya O.A., Monakhova A.V., Vlodavets D.V. (2022). Experience of Using Gene Replacement Therapy with Zolgensma^®^ (Onasemnogene Abeparvovec) in Real Clinical Practice in Russia. Neuromuscul. Dis..

[70] Bitetti I., Manna M.R., Stella R., Varone A. (2024). Motor and Neurocognitive Profiles of Children with Symptomatic Spinal Muscular Atrophy Type 1 with Two Copies of SMN2 before and after Treatment: A Longitudinal Observational Study. Front. Neurol..

[71] Mendonça R., Jorge Polido G., Ciro M., Solla D.J.F., Reed U.C., Zanoteli E. (2021). Clinical Outcomes in Patients with Spinal Muscular Atrophy Type 1 Treated with Nusinersen. J. Neuromuscul. Dis..

[72] D’Silva A.M., Holland S., Kariyawasam D., Herbert K., Barclay P., Cairns A., MacLennan S.C., Ryan M.M., Sampaio H., Smith N. (2022). Onasemnogene Abeparvovec in Spinal Muscular Atrophy: An Australian Experience of Safety and Efficacy. Ann. Clin. Transl. Neurol..

[73] Favia M., Tarantino D., Cerbo L.D., Sabia A., Campopiano R., Pani M. (2024). Onasemnogene Abeparvovec: Post-Infusion Efficacy and Safety in Patients with Spinal Muscular Atrophy (SMA)-A Fondazione Policlinico Gemelli IRCCS Experience. Hosp. Pharm..

[74] Lavie M., Rochman M., Domany K.A., Tripto I.G., Be’er M., Besor O., Sagi L., Aharoni S., Ginsberg M., Noyman I. (2024). Respiratory Outcomes of Onasemnogene Abeparvovec Treatment for Spinal Muscular Atrophy: National Real-World Cohort Study. Eur. J. Pediatr..

[75] McMillan H.J., Baranello G., Farrar M.A., Zaidman C.M., Moreno T., De Waele L., Jong Y.-J., Laugel V., Quijano-Roy S., Mercuri E. (2025). Safety and Efficacy of IV Onasemnogene Abeparvovec for Pediatric Patients with Spinal Muscular Atrophy. Neurology.

[76] Goedeker N.L., Rogers A., Fisher M., Arya K., Brandsema J.F., Farah H., Farrar M.A., Felker M.V., Gibbons M., Hamid O.A. (2024). Outcomes of Early-Treated Infants with Spinal Muscular Atrophy: A Multicenter, Retrospective Cohort Study. Muscle Nerve.

[77] Latzer I.T., Sagi L., Lavi R., Aharoni S., Bistritzer J., Noyman I., Ginsburg M., Lev-Or A., Sharona K., Yoram N. (2023). Real-Life Outcome after Gene Replacement Therapy for Spinal Muscular Atrophy: A Multicenter Experience. Pediatr. Neurol..

[78] Weiß C., Ziegler A., Becker L.-L., Johannsen J., Brennenstuhl H., Schreiber G., Flotats-Bastardas M., Stoltenburg C., Hartmann H., Illsinger S. (2022). Gene Replacement Therapy with Onasemnogene Abeparvovec in Children with Spinal Muscular Atrophy Aged 24 Months or Younger and Bodyweight up to 15 Kg: An Observational Cohort Study. Lancet Child Adolesc. Health.

[79] Martini M., Vecchione L., Siena S., Tejpar S., Bardelli A. (2011). Targeted Therapies: How Personal Should We Go?. Nat. Rev. Clin. Oncol..

[80] Henderson M.L., Zieba J.K., Li X., Campbell D.B., Williams M.R., Vogt D.L., Bupp C.P., Edgerly Y.M., Rajasekaran S., Hartog N.L. (2024). Gene Therapy for Genetic Syndromes: Understanding the Current State to Guide Future Care. BioTech.

[81] Nishio H., Niba E.T.E., Saito T., Okamoto K., Takeshima Y., Awano H. (2023). Spinal Muscular Atrophy: The Past, Present, and Future of Diagnosis and Treatment. Int. J. Mol. Sci..

[82] Dosi C., Masson R. (2024). The Impact of Three SMN2 Gene Copies on Clinical Characteristics and Effect of Disease-Modifying Treatment in Patients with Spinal Muscular Atrophy: A Systematic Literature Review. Front. Neurol..

[83] Janzen E., Mendoza-Ferreira N., Hosseinibarkooie S., Schneider S., Hupperich K., Tschanz T., Grysko V., Riessland M., Hammerschmidt M., Rigo F. (2018). CHP1 reduction ameliorates spinal muscular atrophy pathology by restoring calcineurin activity and endocytosis. Brain.

[84] Ahmad S., Wang Y., Shaik G.M., Burghes A.H., Gangwani L. (2012). The zinc finger protein ZPR1 is a potential modifier of spinal muscular atrophy. Hum. Mol. Genet..

[85] Cao Y.Y., Qu Y.J., Bai J.L., Jin Y.W., Wang H., Song F. (2013). Correlation of PLS3 Expression with Disease Severity in Children with Spinal Muscular Atrophy. J. Hum. Genet..

[86] Muiños-Bühl A., Rombo R., Ling K.K., Zilio E., Rigo F., Bennett C.F., Wirth B. (2023). Long-Term SMN- and Ncald-ASO Combinatorial Therapy in SMA Mice and NCALD-ASO Treatment in HiPSC-Derived Motor Neurons Show Protective Effects. Int. J. Mol. Sci..

[87] Singh R.N., Ottesen E.W., Singh N.N. (2020). The First Orally Deliverable Small Molecule for the Treatment of Spinal Muscular Atrophy. Neurosci. Insights.

[88] Peddi N.C., Ramesh H.M., Gude S.S., Gude S.S., Vuppalapati S. (2022). Intrauterine Fetal Gene Therapy: Is That the Future and Is That Future Now?. Cureus.

[89] David A.L., Waddington S.N. (2012). Candidate Diseases for Prenatal Gene Therapy. Methods Mol. Biol..

[90] Brown S.M., Ajjarapu A.S., Ramachandra D., Blasco-Pérez L., Costa-Roger M., Tizzano E.F., Sumner C.J., Mathews K.D. (2024). Onasemnogene-Abeparvovec Administration to Premature Infants with Spinal Muscular Atrophy. Ann. Clin. Transl. Neurol..

[91] Finkel R.S., Hughes S.H., Parker J., Civitello M., Lavado A., Mefford H.C., Mueller L., Kletzl H. (2025). Risdiplam for Prenatal Therapy of Spinal Muscular Atrophy. N. Engl. J. Med..

[92] Walter M.C., Chiriboga C., Duong T., Goemans N., Mayhew A., Ouillade L., Oskoui M., Quinlivan R., Vázquez-Costa J.F., Vissing J. (2021). Improving Care and Empowering Adults Living with SMA: A Call to Action in the New Treatment Era. J. Neuromuscul. Dis..

[93] Parsons J., Kuntz N., Brandsema J., Proud C., Finkel R., Swoboda K., Masson R., Foster R., Liu Y., Makepeace C. (2023). Somera-Molina. P210 Interim Results from the RESPOND Study Evaluating Nusinersen in Children with Spinal Muscular Atrophy Previously Treated with Onasemnogene Abeparvovec. Neuromuscul. Disord..

[94] Schorling D.C., Pechmann A., Kirschner J. (2020). Advances in Treatment of Spinal Muscular Atrophy—New Phenotypes, New Challenges, New Implications for Care. J. Neuromuscul. Dis..

[95] Saffari A., Kölker S., Hoffmann G.F., Weiler M., Ziegler A. (2018). Novel challenges in spinal muscular atrophy—How to screen and whom to treat?. Ann. Clin. Transl. Neurol..

[96] Yeo C.J.J., Tizzano E.F., Darras B.T. (2024). Challenges and opportunities in spinal muscular atrophy therapeutics. Lancet Neurol..

[97] Mishra N.K., Mishra A. (2025). Spinal Muscular Atrophy (SMA): Treatment strategies, challenges and future prospects. Pharmacol. Res.-Rep..

[98] Belančić A., Gkrinia E.M.M., Eustaquio P., Faour A.K., Vitezić D. (2025). Switching disease-modifying therapies in patients with spinal muscular atrophy: A systematic review on effectiveness outcomes. Br. J. Clin. Pharmacol..

[99] Kery R., Chen A.P.F., Kirschen G.W. (2020). Genetic Targeting of Astrocytes to Combat Neurodegenerative Disease. Neural Regen. Res..

[100] Saito K., Shigetomi E., Shinozaki Y., Kobayashi K., Parajuli B., Kubota Y., Sakai K., Miyakawa M., Horiuchi H., Nabekura J. (2023). Microglia Sense Astrocyte Dysfunction and Prevent Disease Progression in an Alexander Disease Model. Brain.

[101] Verheijen B.M. (2017). Effects of Astroglia on Motor Neurons in Spinal Muscular Atrophy. J. Neurosci..

[102] Yshii L., Pasciuto E., Bielefeld P., Mascali L., Lemaitre P., Marino M., Dooley J., Kouser L., Verschoren S., Lagou V. (2022). Astrocyte-Targeted Gene Delivery of Interleukin 2 Specifically Increases Brain-Resident Regulatory T Cell Numbers and Protects against Pathological Neuroinflammation. Nat. Immunol..

[103] Vandenbark A.A., Offner H., Matejuk S., Matejuk A. (2021). Microglia and Astrocyte Involvement in Neurodegeneration and Brain Cancer. J. Neuroinflamm..

[104] Hickman S., Izzy S., Sen P., Morsett L., El Khoury J. (2018). Microglia in Neurodegeneration. Nat. Neurosci..

[105] Zhong L., Sheng X., Wang W., Li Y., Zhuo R., Wang K., Zhang L., Hu D.-D., Hong Y., Chen L. (2023). TREM2 Receptor Protects against Complement-Mediated Synaptic Loss by Binding to Complement C1q during Neurodegeneration. Immunity.

[106] Musunuru K., Grandinette S.A., Wang X., Hudson T.R., Briseno K., Berry A.M., Hacker J.L., Hsu A., Silverstein R.A., Hille L.T. (2025). Patient-Specific In Vivo Gene Editing to Treat a Rare Genetic Disease. N. Engl. J. Med..

[107] Haque U.S., Yokota T. (2024). Recent Progress in Gene-Targeting Therapies for Spinal Muscular Atrophy: Promises and Challenges. Genes.

[108] Belančić A., Janković T., Gkrinia E.M.M., Kristić I., Bumber J.R., Rački V., Pilipović K., Vitezić D., Mršić-Pelčić J. (2025). Glial Cells in Spinal Muscular Atrophy: Speculations on Non-Cell-Autonomous Mechanisms and Therapeutic Implications. Neurol. Int..

